# Proceedings of the 15th annual conference of INEBRIA

**DOI:** 10.1186/s13722-018-0121-5

**Published:** 2018-09-27

## A1 Single episode of harmful alcohol use resulting in injury: a missed opportunity for brief intervention in the emergency department

### Cheryl J. Cherpitel^1^, Yu Ye^1^, Vladimir B. Poznyak^2^

#### ^1^Public Health Institute, Alcohol Research Group, Emeryville, CA, USA; ^2^Department of Mental Health and Substance Abuse, World Health Organization, Geneva, Switzerland

##### **Correspondence:** Cheryl J. Cherpitel (ccherpitel@arg.org)

*Addiction Science & Clinical Practice* 2018, **13(Suppl 1)**: A1

**Background:** The aim of this paper is to evaluate the extent to which injured patients admitted to the emergency department (ED) with an alcohol-related injury may go undetected if signs of intoxication or withdrawal, a positive blood alcohol concentration (BAC), or signs of alcohol dependence or harmful drinking are relied upon as indicators of alcohol involvement in the injury event.

**Materials and methods:** An alcohol-related injury (drinking within six hours prior to the injury event and causal attribution of injury to drinking) is examined by the amount consumed, BAC and usual drinking pattern for those with and without alcohol dependence or harmful drinking in a representative sample of 18,369 injured ED patients in 23 countries.

**Results:** 18.8% reported drinking in the six hours prior to injury, and 47.1% of these attributed a causal association of their injury to drinking. 16.3% of those reporting drinking and 10.3% of those attributing a causal association were negative for dependence or harmful drinking. The vast majority of both groups reported no heavy (5 +) drinking occasions during the last year and had a BAC of < 0.05 or negative. About a third of both groups were female and most were aged 50 and younger.

**Conclusions:** Findings here suggest that some individuals may have an alcohol-related injury due to a single episode of drinking without a history of harmful use or dependence, and underscores the clinical and public health relevance of screening for these individuals in the ED to identify those who may benefit from a brief intervention. These findings also underscore the utility of a new diagnostic category of a single episode of harmful drinking proposed in the 11th revision of the International Classification of Disease.

## A2 Social workers’ and their clients’ attitudes toward alcohol screening and counselling

### Elina Renko

#### Department of Social Sciences, University of Helsinki, Helsinki, Finland

##### **Correspondence:** Elina Renko (elina.renko@helsinki.fi)

*Addiction Science & Clinical Practice* 2018, **13(Suppl 1)**: A2

**Background**: Social work professionals frequently encounter clients with alcohol-related problems and thus can play a central role in the early identification of problems. However, there has been little research on how professionals or their clients perceive alcohol screening and counselling. This study explores the topic and presents a qualitative analysis of social workers’ and their clients’ attitudes toward alcohol screening and counselling. The analytical focus is on how the two parties constructed screening and counselling in their arguments and did professionals and clients do this in the same way or were there differences between them?

**Materials and methods:** The study employs a qualitative attitude approach. The aim of the approach is to explore the construction of attitudes in argumentative talk. Social work professionals (N = 14) and their clients (N = 14) were asked to comment on the eight statements concerning identification and management of alcohol-related problems. The analysis was performed in two stages. In the classifying analysis, different types of stands or justifications towards each statement were identified. Then, the interpretative analysis brought categories into a conceptual dialogue with relevant theoretical concepts and discussions. Here, the primary objective is to explore how alcohol screening and counselling were constructed as attitude objects.

**Results:** Analysis of the qualitative data reveals that both professionals and clients constructed alcohol screening and counselling similarly as: (1) useful tools for motivation, (2) self-evident parts of social work and (3) tools for discussing sensitive topics. However, compared with the clients, the professionals appeared to associate alcohol screening and counselling more closely with the client’s fulfilment of responsibilities and the ability to function well. On the other hand, compared with the professionals, the clients connected alcohol screening and counselling more closely to privacy-threatening interaction.

**Conclusions:** The professionals and their clients appeared to have common ways to construct alcohol screening and counselling. However, the professionals focused on the client’s responsibilities and well-being; the clients place the same emphasis on interaction and discussion about privacy-threatening topics.

## A3 Cessation of injection drug use following brief assessment interventions for young adults

### Steven P. Kurtz, Mance E. Buttram

#### Center for Applied Research on Substance Use and Health Disparities, Department of Justice and Human Services, Nova Southeastern University, Miami, FL, USA

##### **Correspondence**: Steven P. Kurtz (steven.kurtz@nova.edu)

*Addiction Science & Clinical Practice* 2018, **13(Suppl 1)**: A3

**Background:** The opioid epidemic in the US has severely impacted young adults, and threatens to reverse prior successes in reducing HIV and HCV transmission among people who inject drugs (PWID). We conducted subgroup analyses examining injection cessation among participants in a behavioral intervention trial designed for young adults who use drugs in the nightclub scene.

**Materials and methods:** Participants were enrolled in a 3-arm randomized trial testing the efficacy of age peer interviewer- or self-administered health and social risk assessments compared to waitlist control; outcomes were measured at 12 months. Eligibility criteria included ages 18–39, recent heterosexual risk behavior and multidrug use. Assessments queried demographics, social support and resilience; infectious disease and victimization histories; and prescription (Rx) and illicit drug use. Subanalyses included participants reporting recent IDU at baseline who also completed the 12-month follow-up interview (N = 69). Chi square and F statistics examined differences between participants reporting discontinuing IDU at follow-up compared to those with ongoing IDU.

**Results**: Median age = 25; 33% female; 70% Hispanic, 23% White, 6% Black and 1% other race/ethnicity. Education: 58% some college or more. Childhood victimization history: 44%. Infectious disease prevalence: HIV 3%, HCV 16%. Recent substance use: greater than 90% marijuana, cocaine, heroin, Rx opioids and benzodiazepines; 61% methamphetamine; 58% LSD; and 55% Rx stimulants. Cessation of IDU at 12-month follow-up was reported by 54% of the sample (N = 37). Cessation was unrelated to demographics or baseline substance use frequencies. PWID exposed to the interviewer-administered intervention were more likely to report IDU cessation compared to other conditions (p = .008). Cessation was less likely among PWID who injected both stimulants and opioids at baseline (p = .004), as well as among those with childhood victimization histories (p = .003). Indices of social support, resilience, and positive coping behaviors were higher among PWID reporting injection cessation (all p < .01).

**Conclusions:** The efficacy of the interviewer-administered intervention in reducing IDU was high and similar to the findings for overall drug use reduction in the larger study. PWID with histories of childhood victimization, who score low on measures of resilience, and/or who inject multiple substances appear to require more intensive intervention approaches.

## A4 Perceived motivation as a moderator of the effectiveness of a web-based brief intervention among college drinkers: a four-arm pragmatic controlled trial

### Andre Bedendo^1^, André L. M. Andrade^2^, Maria Lucia O. Souza-Formigoni^3^, Ana R. Noto^1^

#### ^1^Núcleo de Estudos e Pesquisa em Saúde e Uso de Substâncias (NEPSIS), Departamento de Psicobiologia, Escola Paulista de Medicina, Universidade Federal de Sao Paulo, Sao Paulo, Brazil; ^2^Departamento de Psicologia, Pontifícia Universidade Católica de Campinas, Campinas, Brazil; ^3^Unidade de Dependência de Drogas (UDED), Departamento de Psicobiologia, Escola Paulista de Medicina, Universidade Federal de Sao Paulo, Sao Paulo, Brazil

##### **Correspondence**: Andre Bedendo (andrebedendo@gmail.com)

*Addiction Science & Clinical Practice* 2018, **13(Suppl 1)**: A4

**Background:** Web-based *Personalized Normative Feedback* (PNF) interventions are effective in reducing alcohol use among college students, but its efficacy may be affected by participants’ interest and motivation to receive the intervention. This study aimed at evaluating the effectiveness of a web-based PNF and its components, *Normative Feedback* (NF) and *Consequences Feedback* (CF), in reducing alcohol use and negative consequences among college students with different motivation levels for receiving the intervention.

**Materials and methods:** College students aged 18–30 years and alcohol use during the last 3 months recruited through a site developed by a partnership between NEPSIS and Centro de Integração Empresa-Escola. Participants were randomized into four groups: (1) Control Group (CG): assessment-only; (2) PNF; (3) NF and (4) CF. They were followed-up after one (T1), three (T2) and six (T3) months. Students were categorized as low motivated (scores < 3) or motivated (scores ≥ 3) using a visual analogue scale. Outcomes were: AUDIT scores; the number of consequences; and the number of typical drinks. We used Generalized Linear Mixed Models considering the attrition during the follow-up (Multiple Imputation and Pattern-Mixed Model).

**Results:** 8,211 students completed at least one follow-up. PNF was more effective in reducing the typical drinks at T1 and T2, compared to CG. These effects were higher among motivated students. Low motivated participants who received PNF increased AUDIT scores at T3 significantly. At T1, the NF group presented lower AUDIT scores and fewer consequences than the PNF group. The CF group presentea d reduction in AUDIT scores at T2. When considered only motivated participants, NF group reduced consequences, but increased typical drinks at T2 compared with PNF. Among low motivated students, the NF reduced the number of consequences at T1, while the CF group reduced AUDIT score at T2.

**Conclusions**: PNF and both components (NF and CF) reduced only alcohol use while NF also reduced alcohol-related consequences. Motivation moderated the intervention effects, with PNF and NF showing negative effects among low motivated students. CF reduced alcohol use among both low motivated and motivated participants.

**Clinical Trials Identifier:** NCT02058355.

## A5 Correlates of pregnant women’s participation in substance use screening integrated into prenatal care

### Kelly C. Young-Wolff^1^, Lue-Yen Tucker^1^, Mary Anne Armstrong^1^, Amy Conway^2^, Constance Weisner^3^, Nancy Goler^4^

#### ^1^Division of Research, Kaiser Permanente Northern California, Oakland, CA, USA; ^2^Early Start Program, Kaiser Permanente Northern California, Oakland CA, USA; ^3^Department of Psychiatry, University of California, San Francisco, CA, USA; ^4^Regional Offices, Kaiser Permanente Northern California, Oakland CA, USA

##### **Correspondence**: Kelly C. Young-Wolff (kelly.c.young-wolff@Kp.org)

*Addiction Science & Clinical Practice* 2018, **13(Suppl 1)**: A5

**Background:** Kaiser Permanente Northern California (KPNC)’s Early Start program provides universal substance use screening by toxicology and self-report as part of standard prenatal care. Women who screen positive are referred for in-depth substance use screening with an Early Start specialist linked to their prenatal care. This study aimed to examinthe e demographic and clinical correlates of participation in this in-depth screening integrated into prenatal care in a large healthcare system.

**Materials and methods:** The sample comprised KPNC pregnant women with a live birth in 2014 or 2015, who screened positive for substance use in pregnancy via a self-reported questionnaire or a urine toxicology test given as part of standard prenatal care (at ~ 8 weeks gestation). Multivariable logistic regression identified factors related to receiving in-depth substance use screening.

**Results:** There were 11,843 women in the study sample (median [IQR] age, 30 [25–34] years; 42% white; 52% used alcohol, 31% marijuana, 27% other drugs, and 15% nicotine). Of those, 9,836 (83%) completed the in-depth screening. Younger age, lower income, substance use screening in the first trimester, lower parity, single marital status, alcohol use, marijuana use, and nicotine use, and having a psychiatric or substance use disorder or positive toxicology test were associated with higher odds of screening completion (ps < .05).

**Conclusions:** Results provide novel data to enhance understanding of barriers to substance use screening embedded within prenatal care in a real-world clinical setting. Findings suggest that pregnant women from disparity groups are more likely to engage in in-depth substance use screening. Future research is needed to better understand whether differences in screening are due to patient factors (e.g., stigma, availability of other resources) or provider factors (e.g., working harder to engage patients from disparity groups).

## A6 A systematic review of interventions to reduce alcohol use among university students

### Maria Claudia Rodrigues, Erikson F. Furtado

#### Department of Neurosciences and Behavioral Sciences, University of São Paulo, Sao Paulo, Brazil

##### **Correspondence**: Maria Claudia Rodrigues (mariac_rodrigues@yahoo.com.br)

*Addiction Science & Clinical Practice* 2018, **13(Suppl 1)**: A6

**Background:** There is a growing number of studies that point to the excessive use of alcohol within the university environment. Many studies report that preventive interventions are needed to help students to reduce alcohol consumption and its associated risk behaviors. The objective of this study was to systematically review interventions designed to reduce excessive alcohol consumption among university students and to critically analyze the methodology employed.

**Materials and methods:** A systematic review was performed using the PubMed, Web of Science, Scopus, Lilacs and Scielo databases and thkeywordsds: intervention (motivational OR prevention); alcohol (binge OR drinking); college students (university OR universities OR college). We included studies published between 2012 and 2016, which performed and/or evaluated an intervention to reduce alcohol consumption in university undergraduate students; using samples of both sexes and in any age group.

**Results:** 27 studies were included in the review. The sample size of the studies ranged from 32 to 3422 (average = 278), and the mean age was 20.8 years. Regarding measurement instruments, 12 studies used the Alcohol Use Disorder Identification Test (AUDIT) and 11 studies used the Daily Drinking Questionnaire (DDQ). All the studies aimed to analyze the effects and/or effectiveness of Brief Intervention (BI), and a majority (18 out of 27 studies) were effective in reducing alcohol consumption in university students; no study showed an increase in consumption for the intervention groups. We divided the studies into two groups, according to the intervention type: Group 1 (n = 19)—Intervention via the internet (70.4%) and Group 2 (n = 8)—Face-to-face intervention (26.6%). In group 1, eleven studies presented a significant reduction in alcohol consumption among college students (57.9%); while in group 2, seven studies presented a significant reduction (87.5%). The data varied greatly in relation to the duration and content of the interventions, and the duration of the follow-up period.

**Conclusions:** In general, BI is a favorable method for reducing excessive alcohol consumption in the university environment, with proportional data suggesting a greater reduction in the quantity and/or frequency of alcohol consumption for face-to-face interventions.

## A7 A multi-site patient randomized controlled trial of computer-facilitated screening and clinician brief advice for adolescent primary care patients

### Sion K. Harris^1,2^, Lon Sherritt^3^, Laura Grubb^4^, Ronald Samuels^5^, Thomas Silva^6^, Louis Vernacchio^7^, Wendy Wornham^8^, John R. Knight^9^

#### ^1^Harvard Medical School, Boston, MA, USA; ^2^Center for Adolescent Substance Abuse Research, Boston Children’s Hospital, Boston, MA, USA; ^3^Division of Adolescent/Young Adult Medicine, Boston Children’s Hospital, Boston, MA, USA; ^4^Department of Pediatrics, Tufts Medical School, Boston, MA, USA; ^5^Division of General Pediatrics, Boston Children’s Hospital, Boston, MA, USA; ^6^East Boston Neighborhood Health Center, East Boston, MA, USA; ^7^Longwood Pediatrics, Boston, MA, 02115, USA; ^8^Lexington Pediatrics, Lexington, MA, 02421, USA; ^9^Departments of Medicine and Psychiatry, Boston Children’s Hospital, Boston, MA, USA

##### **Correspondence**: Sion K. Harris (sion.harris@childrens.harvard.edu)

*Addiction Science & Clinical Practice* 2018, **13(Suppl 1)**: A7

**Background:** Professional guidelines recommend primary care SU screening and brief advice for all adolescents, but studies show suboptimal adherence. To reduce key barriers, we developed a computer-facilitated Screening and Brief Advice (cSBA) system consisting of computerized pre-visit screening and psychoeducation for patients, and point-of-care decision support for clinicians with prompts to guide 2–3 min of counseling and recommendea d follow-up. We conducted an initial patient randomized controlled trial of cSBA versus treatment as usual (TAU) to test effects on adolescent receipt of clinician advice to avoid SU, and on SU prevention during 12-months follow-up as indicated by time to first SU post-visit.

**Materials and methods:** Well-visit patients ages 12–18 years were consecutively recruited at 5 pediatric offices in Boston in 2015–2016. Participants provided informed assent/consent and completed the CRAFFT 2.0 screen and baseline assessments on a tablet computer prior to the clinician encounter. We had an IRB-approved waiver of parent consent. Participants were then randomized within site (1:2.5) to TAU (n = 243) or cSBA (n = 624). We assessed patient-reported advice receipt with a post-visit questionnaire, and substance use days at baseline and through 12-months follow-up using a Timeline Follow-Back calendar-based method completed confidentially online or by phone at 3-month intervals. We used Cox proportional hazards modeling to compare days-to-first-use post-visit, controlling for age.

**Results:** Participation was 89% (869/1062); 79% of participants had data at 12 months follow-up, with no differences in retention between groups. We found no baseline group differences other than age, with mean age older in TAU vs. cSBA (*M *= 15.1 vs. 14.7 years, p < .05); age was a control variable in all further analyses. The total sample had 51% girls, 45% were White non-Hispanic, and 65% had college- graduate parents. Most (78%) had > 3 prior visits with their clinician. Baseline past-12-month alcohol, cannabis, and other drug use rates were 22, 12, and 1%, respectively. cSBA had higher advice rates compared to TAU (90% vs. 72%, p < .001), and longer time to first post- visit substance use, as indicated by adjusted hazard ratios (AHR) of .77 (95%CI .61–.98) for any substance, .75 (.59–.96) for alcohol, and .61 (.44–.86) for cannabis.

**Conclusions:** Computer-facilitated adolescent screening and clinician brief advice significantly delayed, compared to usual care, time to first substance use following the pediatric well-visit.

## A8 Predictors of engagement, response to follow-up and extent of alcohol reduction in users of a smartphone app, Drink Less

### Claire V. Garnett^1^, Olga Perski^2^, Ildiko Tombor^1^, Robert West^1^, Susan Michie^2^, Jamie Brown^1^

#### ^1^Department of Behavioural Science and Health, University College London, London, UK; ^2^Department of Clinical, Education and Health Psychology, University College London, London, UK

##### **Correspondence**: Claire V. Garnett (c.garnett@ucl.ac.uk)

*Addiction Science & Clinical Practice* 2018, **13(Suppl 1)**: A8

**Background:** Digital interventions for alcohol can help achieve reductions in hazardous and harmful alcohol consumption, and potentially have a broader reach than brief interventions delivered by healthcare workers. The Drink Less app for excessive drinkers in the UK was developed using evidence and theory, and a factorial experiment suggested that four of its intervention modules may assist with drinking reduction. However, low engagement and low response to follow-up are important barriers to effectiveness. Research is needed to understand what factors influence variation in users’ level of engagement, response to follow-up and extent of alcohol reduction.

**Materials and methods:** Secondary data analysis of a factorial RCT of the Drink Less app. Participants were aged 18 or over, lived in the UK and had an AUDIT > 7 (indicative of excessive drinking). Socio-demographic and drinking characteristics were assessed at baseline. Engagement was assessed in the first month of use (number of sessions, time on app, number of days used, and percentage of available screens viewed). Response to follow-up and extent of alcohol reduction (change in past week consumption) were measured after one month. Associations were assessed using unadjusted and adjusted regression models (linear or logistic, as appropriate).

**Results:** Age and education qualifications (post-16) were positively associated with all engagement outcomes (both B > .02, p < .001). Age, education qualifications (post-16), and gender (female) were positively associated with response to follow-up (all OR > 1.04, p < .016). Engagement outcomes predicted response to follow-up (all OR > 1.02, p < .001), but not the extent of alcohol reduction (all -.14.070). Baseline drinking characteristics were the only variables associated with the extent of alcohol reduction amongst those followed-up (all B > .49, p < .001).

**Conclusions**: Users of the alcohol reduction app, Drink Less, who were older and had post-16 education qualifications engaged more and were more likely to respond at one-month follow-up. Higher baseline alcohol consumption predicted a greater extent of alcohol reduction amongst those followed-up. Engagement was not associated with the extent of alcohol reduction, indicating that there is not an overall dose–response effect for the Drink Less app and, also considering the factorial experiment results, that exposure to particular modules is more important than intervention dose.

## A9 Integrated stepped care to address alcohol use among patients living with HIV and alcohol use disorder: Results from a randomized clinical trial

### E. Jennifer Edelman^1^, Stephen Aa Maisto^2^, Nathan B. Hansen^3^, Christopher J. Cutter^4^, James Dziura^5^, Yanhong Deng^5^, Lynn E. Fiellin^1^, Patrick G. O’Connor^1^, Roger Bedimo^6^, Cynthia Gibert^7^, Vincent C. Marconi^8^, David Rimland^8^, Maria C. Rodriguez-Barradas ^9^, Michael S. Simberkoff^10^, Amy C. Justice^11^, Kendall J. Bryant^12^, David A Fiellin^1^

#### ^1^Department of Internal Medicine (General Medicine) and Center for Interdisciplinary Research on AIDS, Yale University School of Medicine, New Haven, CT, USA; ^2^Department of Psychology, Syracuse University, Syracuse, NY, USA; ^3^Department of Health Promotion and Behavior, University of Georgia College of Public Health, Athens, GA, USA; ^4^Child Study Center, Yale University School of Medicine, New Haven, CT, USA; ^5^Yale Center for Analytical Sciences, Yale University School of Public Health, New Haven, CT, USA; ^6^Department of Infectious Diseases, Veterans Affairs North Texas Healthcare System and University of Texas Southwestern, Dallas, TX, USA; ^7^Department of Infectious Diseases, Washington DC Veterans Affairs Medical Center and George Washington University School of Medicine and Health Sciences, Washington DC, USA; ^8^Department of Infectious Diseases, Atlanta Veterans Affairs Medical Center and Emory University School of Medicine, Decatur, GA, USA; ^9^Department of Infectious Diseases, Michael E. DeBakey Veterans Affairs Medical Center and Baylor College of Medicine, Houston, TX, USA; ^10^Department of Infectious Diseases, Veterans Affairs New York Harbor Healthcare System and New York University School of Medicine, New York, NY, USA; ^11^Department of Internal Medicine, VA Connecticut Healthcare System and Yale University School of Medicine, West Haven, CT, USA; ^12^National Institute on Alcohol Abuse and Alcoholism HIV/AIDS Program, Bethesda, MD, USA

##### **Correspondence**: E. Jennifer Edelman (ejennifer.edelman@yale.edu)

*Addiction Science & Clinical Practice* 2018, **13(Suppl 1)**: A9

**Background:** Among patients living with HIV (PLWH) with alcohol use disorder (AUD), we sought to examine the effectiveness of integrated stepped care (ISC) versus referral as indicated (RAI) for alcohol use.

**Materials and methods:** We conducted a randomized clinical trial between January 28, 2013–July 14, 2017 in five Veterans Health Administration Infectious Disease Clinics and enrolled PLWH having AUD. ISC over 24 weeks involved: Addiction Physician management with consideration of alcohol treatment medications (Step 1), Motivational Enhancement Therapy (Step 2), and referral for specialty care (Step 3) if patients reported ongoing heavy alcohol use at weeks 4 and 12, respectively. RAI: referral to specialty care at discretion of HIV provider. Primary outcome: average drinks per week (by Timeline Followback). Secondary outcomes: any heavy drinking daysphosphatidyl ethanolol (PEth) level, VACS Index score, undetectable HIV viral load, and depression (Patient Health Questionaire-9). Outcomes were assessed at week 24.

**Results:** Among 128 participants, the mean age was 54 years, 98% were men, and 79% were black. Among those randomized to ISC (n = 63), 47% were stepped up to Step 2 and 25% were stepped up to Step 3. Forty-one percent of ISC patients vs. 8% of RAI patients received alcohol treatment medications. The ISC group reported non-statistically significant fewer average drinks per week compared to RAI group (10.48 vs 18.52, adjusted mean difference [AMD] [95% CI] = − 4.20 [− 9.55, 1.15]) and were less likely to report any heavy drinking days than RAI group (27.27% vs. 54.76%; adjusted odds ratio [AOR] [95% CI] = 0.28 [0.09, 0.89]). ISC group had non-significantly lower mean PEth values than RAI group (151.32 vs. 259.83 ng/mL, AMD [95% CI] = − 49.51 [− 178.11, 79.08]). We were unable to detect differences between ISC and RAI groups on VACS Index scores (27.13 vs. 29.05, AMD of − 1.4 [− 5.0, 2.3]), proportion with an undetectable HIV viral load (68.42% vs. 62.86%, AOR [95% CI] = 1.0 [0.29, 3.60]), and proportion with depression (16.28% vs. 20.93%; AOR [95% CI] = 0.58 [0.12, 2.82]).

**Conclusions:** The reduction in drinks per week in the ISC group did not meet statistical significance, but ISC reduced self-report of heavy alcohol use. Strategies to expand and augment ISC should be considered.

**Trial Registration:** Clinicaltrials.gov NCT01410123.

## A10 Systematic review into the effects of control group changes in interpretation of findings in Alcohol Brief Interventions

### Michael E. Jecks^1^, Matt Field^1^, Caryl Beynon^2^, Mark Gabbay^3^

#### ^1^Department of Psychological Sciences, University of Liverpool, Liverpool, UK; ^2^Risk Factor Intelligence, Public Health England, Liverpool, UK; ^3^Health Services Research, University of Liverpool, Liverpool, UK

##### **Correspondence**: Michael E. Jecks (mjecks@liverpool.ac.uk)

*Addiction Science & Clinical Practice* 2018, **13(Suppl 1)**: A10

Alcohol Brief Interventions (ABI) have been shown to be an effective method of reducing alcohol consumption in non-clinical populations. They are cost-effectiveve, single session intervention which can be deployed among a large population quickly and easily, including by professionals without specific clinical training. However, the reported effect size is usually quite small (d = 0.15) and, worryingly, significant changes in control group drinking regularly occur in ABI Randomised Control Trials. However, it is not yet understood why such changes are found in control groups, or the potential implications this could cause in the literature. A number of possibilities have been suggested, including regression to the mean, demand characteristics (both context effects and hypothesis guessing), assessment reactivity, and the possibility that the control group interventions also include active behaviour change techniques. Without knowing why changes are found in the control groups, we cannot justifiably compare control and intervention groups, because we may be inadvertently comparing ABI with another unintentional intervention. Here, we discuss findings from an exploratory meta-analysis investigating the degree to which control groups are decreasing their drinking and potential factors which may explain this. The meta-analysis included 73 studies into Alcohol Brief Interventions. The meta-analysis shows a significant within-subject decrease in drinking in both the control groups (SMD = 0.27, p < 0.05, 95% CI [0.17, 0.37]) and the intervention groups (SMD = 0.41, p < 0.05, 95% CI [0.31, 0.50), with no significant difference between the groups in terms of the degree of change. This suggests evidence of control groups masking the true intervention effect size. Further sub-group analyses and the implications are discussed.

## A11 Effectiveness of a brief intervention for marijuana users in Mexico

### César Carrascoza Venegas^1^, Leticia Echeverria San Vicente^2^, Miguel Ángel Medina^2^

#### ^1^Facultad de Estudios Superiores Iztacala, Universidad Nacional Autónoma de México, Tlalnepantla, México; ^2^Facultad de Psicología, Universidad Nacional Autónoma de México, Ciudad de México, México

##### **Correspondence**: César Carrascoza Venegas (cesarcarrascoza@hotmail.com)

*Addiction Science & Clinical Practice* 2018, **13(Suppl 1)**: A11

**Background:** In Mexico, marijuana is the most consumed illegal drug, accounting for 80% of the total illegal drug consumption, and in the last survey it went from 6% to 8.6% in 2016 (ENCODAT 2016–2017), this is a problem due to its effects on health, family and escalation to other drugs, in addition to the perception that it is a little dangerous substance. Therefore, it is necessary to develop effective treatments, based on scientific evidence, specifically for this substance. The objective of the work is to describe the application and analyze the results of a Brief Intervention to assist users of marijuana.

**Materials and methods:** The sample was integrated by 52 users of marijuana users with an average age of 22 years, who volunteered to receive treatment in an addiction clinic belonging to the National University of Mexico. A design n = 1 with several replicas was used, to ensure its internal and external validity, a pre, post evaluation and six-month follow-up was done.

**Results:** Statistically significant differences are presented between the initial evaluation and the 6-month follow-up (F = 54.67, P < 0.000).

**Conclusions:** From the results it is emphasized: the importance of using treatments based on scientific evidence; the achievement of abstinence and its maintenance six months after concluding the brief intervention; the use of motivational strategies to sensitize young people and increase the perception of risk on this substance; and Brief intervention as an appropriate alternative to be implemented in public health services in Mexico.

## A12 Brief intervention in school-based health centers: a study of nurse practitioner-delivered vs. computer-delivered BI

### Jan Gryczynski, Robert P. Schwartz, Shannon Gwin Mitchell, Kristi Dusek

#### Friends Research Institute, Baltimore Maryland USA, Baltimore, MD, USA

##### **Correspondence**: Jan Gryczynski (jgryczynski@friendsresearch.org)

*Addiction Science & Clinical Practice* 2018, **13(Suppl 1)**: A12

**Background**: School-based health centers (SBHCs) play an increasingly prominent role in improving healthcare access for adolescents in the US, particularly in underserved communities. SBHCs offer a range of health services, including care for acute and chronic medical problems, disease screening, family planning services, immunizations, and other primary care. Because they are embedded within schools, SBHCs are promising settings for a brief intervention. However, little is known about how best to provide brief interventions in these settings.

**Materials and methods:** A randomized trial comparing the effectiveness of nurse practitioner-delivered brief intervention (NBI) vs. computer-delivered brief intervention (CBI) was conducted in two urban SBHCs. Participants were 300 high school students ages 14–18 with risky alcohol and/or cannabis use as determined by CRAFFT screening. Both interventions were tailored to participant characteristics and behaviors, incorporated motivational interviewing principles, and focused on cannabis, alcohol, and sex risks. Assessments were conducted at baseline, 3- and 6-month follow-up using audio computer-assisted self-interviewing methods. Qualitative semi-structured interviews were conducted with 14 adolescents prior to the launch of the trial (to inform intervention development), 30 adolescent trial participants, and two nurse practitioners. Qualitative interviews were recorded and transcribed for analysis.

**Results:** Participants were 54% female, 93% black, and 6% Hispanic, with a mean age of 16.3 years (SD = 1.1). Past year cannabis and alcohol use were reported by 93% and 67%, respectively, with a mean CRAFFT score of 3.3 (SD = 1.2). Seventy-eight percent used cannabis in the past 30 days (mean = 9.9 of 30 days; SD = 11.1). Past month alcohol use was reported by 40% (mean = 1.2 of 30 days; SD = 2.9). Follow-ups are currently underway and will be completed in August 2018.

**Conclusions:** This presentation will describe findings comparing CBI and NBI, and will examine considerations for deploying these brief intervention strategies in SBHC settings.

## A13 Adherence in a web-based intervention for college drinkers: influence of the participants’ characteristics and recruitment strategies

### Marcella Ferreira Gonçalves, André Bedendo, Ana R. Noto

#### Núcleo de Estudos e Pesquisa em Saúde e Uso de Substâncias (NEPSIS), Disciplina de Medicina e Sociologia do Abuso de Drogas (DIMESAD), Departamento de Psicobiologia, Escola Paulista de Medicina, Universidade Federal de Sao Paulo, Sao Paulo, Brazil

##### **Correspondence:** Marcella Ferreira Gonçalves (marcellafg@tjsp.jus.br)

*Addiction Science & Clinical Practice* 2018, **13(Suppl 1)**: A13

**Background:** Attrition is an important issue in web-based interventions for college drinkers. Previous studies showed that participants’ characteristics are related to attrition, but there is a lack of studies evaluating the influence of different recruitment strategies. This study aims to analyze the influence of the participants’ ´ characteristics and different recruitment strategies in adherence (at least one follow-up assessment) on a web-based intervention for college drinkers.

**Materials and methods:** 46,332 college students from all Brazilian regions aging from 18 to 30 years and reporting alcohol use in the previous three months. Students were recruited using non-incentive strategies (email invitations and Facebook) or incentives (academic credits). Participants were followed after 1, 3 and 6 months. The questionnaire included educational and sociodemographic characteristics, alcohol use (AUDIT), alcohol-related consequences and motivation to know more about their alcohol consumption. Statistical analyzes considered logistic regression models adjusted for sex, age, income and region.

**Results:** Women (aOR = 1.09 95% CI: 1.03; 1.17 p = 0.005) and students with higher socioeconomic status (aOR = 1.38 95% CI: 1.23; 1.55 p < 0.001) were more adherent. More motivated students showed higher the adherence (aOR = 1.55 95% CI: 1.43; 1.69 p < 0.001). Regarding alcohol use, binge drinkers were more likely do adhere than non-binge drinkers (aOR = 1.23 95% CI: 1.15; 1.32 p < 0.001), as well as participants with alcohol hazardous use (aOR = 1.18 95% CI: 1.09; 1.28 p < 0.001), compared to low-risk drinkers. In contrast, participants reporting more alcohol-related consequences, were less adherent (aOR = 0.54 95% CI: 0.33; 0.91 p < 0.001). Recruitment using incentives increased by 5 times the adherence (aOR = 5.62 95% CI: 4.31; 7.34 p < 0.001), compared to non-incentives.

**Conclusions:** The participants’ characteristics interfered with adherence in a web-based intervention. Male, lower socioeconomic level, and negative consequences reduced adherence, while binge drinking, hazardous alcohol use, more motivated and non-monetary incentive increased adherence. This study highlights subgroups of participants that are more adherent and the impact of different recruitment strategies over attrition. Findings may help to improve future web-based alcohol interventions.

**Clinical Trials Identifier:** NCT02058355.

## A14 Proactive health risk screening for multiple e-health interventions in primary care patients: methods, design and reach

### Anja Bischof^1^, Diana Guertler^2^, Kristian Krause^2^, Anne Moehring^2^, Gallus Bischof^1^, Hans-Juergen Rumpf^1^, Ulrich John^2^, Christian Meyer^2^

#### ^1^Department of Psychiatry and Psychotherapy, University of Luebeck, Luebeck, Germany; ^2^Institute of Social Medicine and Prevention, University Medicine of Greifswald, Greifswald, Germany

##### **Correspondence:** Anja Bischof (anja.bischof@uksh.de)

*Addiction Science & Clinical Practice* 2018, **13(Suppl 1)**: A14

**Background:** General practices and general hospitals are efficient settings for the provision of proactive prevention efforts and E-health interventions and have the potential to serve large populations. However, the cost of proactive recruitment is a factor limiting implementation, especially since most E-health interventions are focused on single behavioral health risks, making systematic screening ineffective. We describe methodological details of a proactive multipurpose health risk screening procedure in primary care patients and report data on the reach of individuals.

**Materials and methods:** Study assistants proactively approached patients between 18 and 64 years from general practices and general hospitals at three sites for a computerized screening on harmful alcohol and tobacco consumption, depressiveness, insufficient fruit/vegetables consumption, physical inactivity, and overweight. An automatic scoring algorithm allocated patients to one of five studies addressing unhealthy substance use and/or depression. We analyzed participation rates, participants’ characteristics, and selection factors.

**Results:** In total, 12,757 patients were screened (overall participation rate: 86%) with 60% reporting at least two health risks. General practice patients reported fewer health risks (1.6 vs. 1.7) but more depressive symptoms (19% vs. 12%) than general hospitals patients. Of all participants screened, 30% were eligible for study participation. Between 36% and 51% of all eligible patients gave informed consent for study participation. Screening participation was associated with age and gender; study participation with socio-demographics, the number of health risks, and risk severity.

**Conclusions:** Combining E-health interventions with proactive recruitment can achieve good reach among users who were not ready to change behavior yet. Since a substantial proportion of patients in primary care reveal multiple health behaviors, screening procedures should simultaneously address various health behaviors. More research is needed on how to increase participation rates and create synergies in E-health interventions for patients presenting multiple health risks.

## A15 AUDIT – psychometric properties to identifying alcohol dependence

### Sara Wallhed Finn, Sven Andréasson

#### Department of Public Health Sciences, Karolinska Institutet, Stockholm, Sweden, Stockholm

##### **Correspondence:** Sara Wallhed Finn (sara.wallhed-finn@ki.se)

*Addiction Science & Clinical Practice* 2018, **13(Suppl 1)**: A15

**Background:** Individuals with alcohol dependence are rarely diagnosed in health care services. This emphasizes the need for questionnaires with good psychometric properties in order to support healthcare staff to identify a larger proportion. A commonly used questionnaire is the AUDIT (Alcohol Use Disorders Identification Test). An important question is the possibility to identify alcohol dependence in clinical populations with AUDIT. Objective: To investigate cut off scores for alcohol dependence in a clinical population, and whether combining AUDIT with biological markers can improve specificity and sensitivity.

**Materials and methods:** Data collected from 498 patients seeking treatment at a specialized addiction clinic in Stockholm 2012–2014 was used for analysis. Patients filled in AUDIT and provided blood specimen, analyzed for carbohydrate-deficient transferrin (CDT), alanine aminotransferase (ALT), aspartate aminotransferase (AST) and gamma-glutamyl transferase (GGT). Analysis was performed with methods from classical test theory; internal consistency, sensitivity, specificity, and ROC. ICD-10 diagnosis by a physician was Gold Standard for alcohol dependence.

**Results:** AUDIT10 showed better sensitivity and specificity for alcohol dependence compared to AUDIT-C, AUDIT-3 and the dependence questions in AUDIT. For AUDIT-10 cut off > 16 gave the best balance between sensitivity (89.7%) and specificity (53.9%) with 78% correctly classified. Among 25–45 years old, > 18 or > 20, gave the best balance between sensitivity and specificity, with ROC area 88%. In the group 46–55 years, cut off > 16 was the best, with ROC 81%. Among 56–80 years, a lower cut off > 14 gave the highest accuracy. However, sensitivity and specificity were lower among the oldest. The lowest ROC area, 72%, was found among older women, significantly lower compared to the youngest group. Cut off > 15 in combination with elevated ALT improved sensitivity and specificity the most. AUDIT combined with elevated CDT, GGT, and AST only slightly improved sensitivity and specificity.

**Conclusions:** AUDIT-10 showed the best psychometric properties to identify alcohol dependence among treatment seekers. A cut off of > 16 gave the highest sensitivity and specificity, which is lower compared to previous studies. Combining AUDIT with ALT improved psychometric properties. With older age, AUDIT shows lower psychometric properties, especially among older women, which future studies should investigate further.

## A16 Standard joint unit: a new tool for assessing risky use

### Eugènia Campeny de Lara^1^, Hugo López-Pelayo^2^, Cristina Casajuana^1^, Joan Colom^3^, Maria Mercedes Balcells^2^, Antoni Gual^1^

#### ^1^Addictions Unit, Department of Psychiatry, Hospital Clínic, Institut d’Investigacions Biomèdiques August Pi i Sunyer (IDIBAPS), Barcelona, Spain; ^2^Addictions Unit, Department of Psychiatry, Clinical Institute of Neuroscience, Hospital Clínic, Fundació Clínic Recerca Biomèdica (FCRB), Barcelona, Spain; ^3^Program on Substance Abuse, Public Health Agency, Government of Catalonia, Barcelona, Spain

##### **Correspondence:** Eugènia Campeny de Lara (campeny@clinic.cat)

*Addiction Science & Clinical Practice* 2018, **13(Suppl 1)**: A16

**Background:** Cannabis is globally the third most consumed drug after alcohol and tobacco. In recent years, the consumption of cannabis has been increasing and, consequently, the number of people demanding treatment has also increased. Despite these trends, a recent systematic review showed that there is no consensus on the definition of risky cannabis use, nor a standard means to define use based on quantity and frequency of consumption. As a response to this situation, we aim to define a Standard Joint Unit (SJU) and develop quantitative criteria to enable screening of risky cannabis use based on the SJU.

**Materials and methods:** A naturalistic study of a convenience sample was made to define the SJU. Adults without cognitive impairment or language barriers completed a questionnaire on their cannabis use during the last 60 days and were asked to donate a joint to determine the 9-THC and Cannabinoid (CBD) content. Socio-demographical data, cannabis quantities, the frequency of use and risk for Cannabis Use Disorder (CUD—measured with the CAST) were collected to establish quantitative criteria to screen for risky cannabis use, based on the SJU. Scores were categorized into low, moderate and high risk of CUD, and related to the number of SJU consumed and frequency on a Receiver Operating Characteristic (ROC) curve.

**Results:** 492 adults reported cannabis use and donated a total of 315 valid joints (232 marijuana; 83 hashish). Participants paid 5€ per gram of cannabis, with which they rolled 4 joints with each joint containing 7 mg of 9-THC (median values). This leads to 1 SJU = 1 joint = 0.25 g of cannabis = 7 mg of 9-THC. The second part of the study looked at risk of CUD among 473 adults, 82.5% of whom consumed cannabis nearly daily and 83.7% more than 4 times per week, with an average of 4 joints per smoking day. Risk for CUD increased significantly with higher frequency and quantity of cannabis use. The strongest predictor was found to be the number of joints consumed per smoking day, suggesting that consuming 1 extra joint a day increases the odds of risk of CUD by 1.44 times (95%, CI 1260–1640). ROC optimal adjustment suggests a cut-off criterion of 1.2 SJU per day to identify moderate/high risk. From this, we suggest 1 SJU per day as a preliminary evidence-based criterion to screen users with at least moderate risk for CUD.

**Conclusions:** Although these results get us nearer to defining a quantitative tool to identify risky cannabis use, there is still a need for early detection and interventions, taking in account the adverse effects of cannabis use on physical and mental health, as well as on social, academic and work functioning. Namely, there is still a lack of definition in harms related to cannabis use. The only available tool is the CAST interview which focuses on the psychiatric disorder parameter. Further investigation is needed to define patterns of risky cannabis use, based on an agreed SJU, in multiple functional dimensions (organic, mental, injury, social, etc.).

## A17 Adolescents’ opinion on psychoactive substances and the relationship with their drug use

### Lilia D’Souza-Li^1^, Gabriela Nogueira Pavan^2^, Luisa Ferreira Roselli^3^

#### ^1^Department of Pediatrics, Faculty of Medical Science, University of Campinas, Campinas, Brazil; ^2^Program of Child and Adolescent Health, Faculty of Health Science, University of Campinas, Campinas, Brazil; ^3^Center for Investigation of Pediatrics, Faculty of Health Science, University of Campinas, Brazil

##### **Correspondence:** Lilia D’Souza-Li (ldesouza@globo.com)

*Addiction Science & Clinical Practice* 2018, **13(Suppl 1)**: A17

**Background**: Understanding what teenagers think about drug use may be the starting point for structuring preventive strategies in order to prevent or delay drug contact. Objective: To evaluate the prevalence of drug use among adolescents and to compare their opinions about drugs with whether or not they use them.

**Materials and methods:** Cross-sectional observational study with patients aged 10–24 years attending a Primary Health Center. A questionnaire containing the CRAFFT and an open question about their opinion on licit and illicit drugs was applied. These responses were divided into two groups: anti-drugs opinion and a pro-drugs or indifferent opinion. Subsequently, all the answers were classified into 7 categories using keywords. To evaluate associations between variables χ2 Test or Fisher’s exact test were used. This project was approved by the Research Ethics Committee.

**Results:** A total of 257 participants, mean age 17 years; 135 (52.5%) 3 18 years old, 188 (73.2%) of whom were female. Of the 253 respondents to CRAFFT, 97 (38.3%) reported use of a substance, 69.1% were female, 35 (26%) were under 18 years of age; 80 patients (83.3%) used alcohol, 30 (30.9%) used cigarettes, 27 (27.8%) used marijuana, 14 (1.6%) used something else, and 39 patients presented a risk behavior for substance use. The use of illicit drugs was similar among minors and those over 18 years old. Of the 211 patients who gave their opinion about drugs, 184 (87.2%) were against drugs, 8 (3.8%) favored some type of substance and 19 (9%) were indifferent. Girls were significantly more against drug use (p = 0.01). A moral discourse was more frequent among those that never used drugs (p = 0.019). Of the 97 adolescents who reported use of a substance, 75% presented an opinion against the drug.

**Conclusions:** Most adolescents and young adults who have used a substance in the last 12 months presented an opinion contrary to drug use. This ambiguity already presented in individuals suggests that they may be more easily convinced to stop using when working and intervening with them on this topic.

## ‬‬‬‬‬A18 Main advantages and disadvantages associated with drinking reported by users of a web-based self-help intervention: influence of gender

### Maria Lucia O. Souza-Formigoni^1^, Fabrício Landi^1^, André L. M. Andrade^2^

#### ^1^Department of Psychobiology, Escola Paulista de Medicina, Universidade Federal de São Paulo, São Paulo, Brazil; ^2^Departamento de Psicologia, Pontifícia Universidade Católica de Campinas, Campinas, Brazil

##### **Correspondence:** Maria Lucia O. Souza-Formigoni (mlformig@gmail.com)

*Addiction Science & Clinical Practice* 2018, **13(Suppl 1)**: A18

**Background**: In partnership with researchers from four countries (Belarus, Brazil, India, and Mexico), the World Health Organization supported the development of an e-health portal on alcohol and alcohol-related consequences, which includes a web-based self-help intervention. The users have access to a set of tools developed to a) prepare for action (know about their alcohol use and find out if they are at risk of having health problems because of drinking); b) set their goals (taking the first step to cut down or stop their drinking) and c) take actions (use their diary to become aware of their drinking amount and related situations; access the step-by-step guide with suggestions to cut-down or stop drinking; do exercises to reflect and feel more positive in order to avoid slipping or relapsing.

**Materials and methods:** We analyzed data from 1317 men and 958 women who entered the site between 2013 and 2018 and reported the main advantages and disadvantages associated with drinking.

**Results:** The main advantages reported were: It is fun to drink (67.7%), I do funny things (51.2%), I make friends more easily (53.6.% men/44.2% women), I feel excited (48%), I feel more relaxed (46.3%). The main disadvantages reported were: I have a hangover (54.5%), I am out of shape (54.2%), I spend too much money (52%), I make a fool of myself (52.5% men and 50.9% women), I do not sleep well (53.1% men/47.8% women). We also observed differences between men and women regarding some less frequently reported advantages (not feeling lonely: 41.5% men/39.7% women) and disadvantages (problems at work 23.9% men/15.8% women; gaining weight: 38% men/46.1% women; reducing sexual performance: 42.5% men/31.4% women and disturbing the relationship with their partners: 35.4% men/25.5% women).

**Conclusions:** The knowledge of the most important pros and cons of drinking, considering gender influence, is helpful to plan strategies to reduce drinking and prevent risk situations.

## A19 Brief intervention for the attention of the harmful use of alcohol: 20 years in Mexico

### Leticia Echeverria San Vicente^1^, César Carrascoza Venegas^2^

#### ^1^Facultad de Psicología, Universidad Nacional Autónoma de México, Ciudad de México, México; ^2^Facultad de Estudios Superiores Iztacala, Universidad Nacional Autónoma de México, Tlalnepantla, México

##### **Correspondence:** Leticia Echeverria San Vicente (echevel@hotmail.com)

*Addiction Science & Clinical Practice* 2018, **13(Suppl 1)**: A19

**Background:** Alcohol is the most commonly used psychoactive substance in the world. In Latin America, it is estimated that the total consumption of alcohol per capita is 30%, higher than the world average. In Mexico, the results of the ENCODAT 2016–2017 show a growth in the tendencies of excessive consumption in the last year, going from 28.0% to 33.6%. In Mexico, since the 1990s, the health sector and other public institutions have made efforts to promote effective alternatives to address this public health problem. The **objective** of this work is to review the results of research conducted in Mexico for several years, applying an intervention for excessive consumers of alcohol. It is a brief motivational, cognitive-behavioral intervention that uses self-control procedures, functional analysis of drinking behavior, identification of risk situations and development of coping strategies.

**Results:** Different groups attended in Mexico City (N = 1028) between 1995 and 2015. Showing changes ranging from 10.75 units of standard drink on average during the pre-evaluation to 4.7 during the intervention and 4.2 during follow-ups of 6 and 12 months, with statistically significant changes by reducing the amount of alcohol consumed, maintaining them through follow-ups, reducing the problems associated with this form of consumption and the increase in the self-efficacy of clients to control episodes of loss of control.

**Conclusions**: The process of dissemination in spaces of the health sector, the barriers and the importance of disseminating the benefits of IB in countries with limited resources such as Mexico are discussed, as they are low-cost options and the possibility to address a public health problem.

## A20 Barriers to alcohol use disorder treatment elicited during a brief intervention

### Kenneth R. Conner^1^, Tracy Stecker^2^, Beau W. Abar^1^, Stephen A. Maisto^3^

#### ^1^Emergency Medicine and Psychiatry, University of Rochester Medical Center, Rochester, NY, USA; ^2^School of Nursing, Medical University of South Carolina, Charleston, SC, USA; ^3^Syracuse University, Syracuse, NY, USA

##### **Correspondence:** Kenneth R. Conner (kenneth_conner@urmc.rochester.edu)

*Addiction Science & Clinical Practice* 2018, **13(Suppl 1)**: A20

**Background:** Individuals who obtain specialty care for alcohol use disorder (AUD) have improved drinking outcomes, yet only a small percentage of individuals with AUD obtain treatment. We sought to identify barriers to AUD treatment seeking among individuals with moderate- to severe AUD symptoms who had never sought treatment.

**Materials and methods:** This is a secondary analysis of a randomized controlled trial, RCT (Stecker T, McGovern M, Herr B, 2012*. An intervention to increase alcohol treatment engagement: A pilot trial. Journal of Substance Abuse Treatment*, 43, 161–167). Participants were age 18-plus, scored ≥ 16 on the Alcohol Use Disorders Identification Test (AUDIT), and had never sought AUD treatment. Intervention arm subjects (N = 99) were administered a one-hour cognitive behavioral intervention by telephone to promote treatment seeking. Study therapists elicited beliefs that served as barriers to treatment seeking that were documented in written therapy notes and were categorized retrospectively.

**Results:** The most common belief was categorized as “afraid of discomfort,” endorsed by 35% of subjects. Examples include: “It is hard to trust someone else.” “I am concerned about withdrawal.” The other beliefs (e.g., “I can control of drinking”) were endorsed by 18% or fewer subjects.

**Conclusions**: With the exception of “afraid of discomfort”, the other barriers to treatment identified have been reported commonly in the literature. Although “afraid of discomfort” has been infrequently mentioned in the literature as a barrier, it was the most commonly endorsed belief in the sample including fear of emotional (e.g., losing control of emotions in a therapy session, reliving trauma, bridging trust) or physical (e.g., craving, withdrawal) discomfort. Moreover, the idea of discomfort associated with treatment was the most emotional and time-consuming belief discussed during sessions. Results suggest the importance of targeting such discomfort in interventions to promote AUD treatment seeking.

## A21 Alcohol screening and brief interventions among homeless and vulnerably housed individuals

### Tereza Maria Barroso^1^, Lisete Cordeiro^2^, Emanuel Pestana^2^, Marina Pereira^3^, Rita Ramos^4^, Antonio Mota^4^

#### ^1^Mental Health Department, Nursing School Coimbra, Coimbra, Portugal; ^2^Associação InPulsar, Leiria, Portugal; ^3^Mental Health Nurse, Unidade de Cuidados à Comunidade Arnaldo Sampaio, Leiria, Portugal; ^4^Escola Superior de Enfermagem de Coimbra, Coimbra, Portugal

##### **Correspondence:** Tereza Maria Barroso (tbarroso@esenfc.pt)

*Addiction Science & Clinical Practice* 2018, **13(Suppl 1)**: A21

**Background:** In Portugal, the alcohol consumption per capita in the adult population exceeds the European mean consumption. The magnitude of this problem among homeless and vulnerably housed individuals (VhI) is unknown. Information about the homeless like how many are there, who are they and why they are in this situation it is very important, but it is also important to know the main health-related behaviors and risks to which they are exposed. The aims of this study were therefore to explore the feasibility and acceptability of an ABI among homeless and VhI, and to develop an ABI to be piloted in a future trial.

**Materials and methods:** Cross-sectional survey, the data were collected with AUDIT, as part of the InPulsar work (Non-Governmental Organization Supporting the Homeless) in a Portuguese city, a sample composed of 32 homeless VhI (mean age 44.8 years, ranges from 26 to 65 years of age), 87,5% are male.

**Results:** 32 Structured interviews were conducted; 6 were foreigners. Only 5 were employed, 12 were unemployed, and 15 receive a pension or income from social insertion. 53.1% reported having already consumed illicit drugs (15.6% are in the methadone program); 68.8% reported alcohol consumption, 34.3% cannabis, 90.6% tobacco, 9.3% hypnotic and sedatives, with regularity. 9 participants were screened positive for hazardous and harmful drinking. A total of 23 education interventions were developed, and 9 brief counseling. All individuals thought to talk about these problems was important and thought that 10 or 20 min of advice would be useful, and agree to be followed-up.

**Conclusion:** Al of homeless and vulnerably housed individuals accepting the screening and brief interventions and would like to participate in follow-up. Education interventions and brief counseling were developed based on the risk level, and the work with the homeless approached these individuals of social support services. We need the follow-up study to analyze the effect of the brief interventions on the reducing hazardous and harmful drinking among vulnerable people.

## A22 Putting the “RT” in SBIRT: an implementation pilot of specialty video consultation in primary care

### Amy S. Leibowitz^1^, Derek D. Satre^1,2^, Constance M. Weisner^1,2^, Stacy A. Sterling^1^

#### ^1^Kaiser Permanente Northern California, Division of Research, Oakland, CA, USA; ^2^Department of Psychiatry, Weill Institute for Neurosciences, University of California, San Francisco, CA, USA

##### **Correspondence:** Amy S. Leibowitz (amy.s.leibowitz@kp.org)

*Addiction Science & Clinical Practice* 2018, **13(Suppl 1)**: A22

**Background**: Brief interventions are known to help primary care (PC) patients reduce unhealthy alcohol use, but there is less evidence about whether screening, brief intervention, and referral to treatment (SBIRT) helps patients with more severe problems connect to addiction treatment. SBIRT holds promise for facilitating successful referral and treatment initiation, but few interventions have addressed the challenges in doing this. We piloted an on-call service whereby specialty addiction medicine consultants provided real-time video consultation—including motivational interviewing and information about psychosocial and medication-assisted treatment options—to patients presenting in PC with likely alcohol use disorders.

**Materials and methods:** Alcohol as a Vital Sign 2.0: Addiction Medicine Video Consultation was a one-year feasibility pilot conducted in adult primary care in a large Kaiser Permanente Northern California (KPNC) medical center. Researchers developed and implemented video consult workflows, continuing medical education (CME) training, an on-call staffing protocol, and ongoing technical assistance. Observational data included CME attendance and consult service utilization rates, technological challenges encountered, and treatment recommendations. Qualitative data included interviews with key stakeholders from addiction medicine, primary care, and information technology. Additional data about patient demographics and outcomes, including specialty and medication-assisted treatment initiation, will be extracted from KPNC electronic health records.

**Results:** Ninety-one of the medical center’s 130 PC physicians (70%) attended training, with 28 (31%) utilizing the consultation service. Eight remotely located addiction medicine consultants provided interventions to PC patients (n = 32). Technological challenges, ranging from a slight lag between audio and video feeds to video failure, occurred in 57% of consults, and 20 attempted consults (38%) did not occur due to technical or staffing problems. Consultants recommended specialty treatment for 23 patients (72%) and anti-craving medication for 13 patients (41%)

**Conclusions:** PC physician engagement and utilization rates, and overall stakeholder feedback suggest that a video consult service would be readily adopted in PC, especially if technology and staffing challenges were addressed. The presentation will also report rates of treatment initiation, medication-assisted treatment prescriptions filled, and lessons learned, which we plan to incorporate into a larger-scale study of patient outcomes and cost-effectiveness.

## A23 Women in primary health care services: patterns of alcohol drinking

### Talita D. Ponce, Erika G. L. Ramirez, Divane de Vargas

#### Department of Mental Health and Psychiatric Nursing, University of São Paulo-School of Nursing, São Paulo, Brazil

##### **Correspondence:** Talita D. Ponce (talitadponce@gmail.com)

*Addiction Science & Clinical Practice* 2018, **13(Suppl 1)**: A23

**Background**: Primary health care services receive women with different patterns of alcohol consumption, but the few studies that have undertaken to investigate this reality include male subjects, making it difficult to investigate the characteristics of alcohol peculiar to the female sex. Therefore, this study aims to identify the pattern of alcohol consumption of women users of Primary Health Care services in the city of São Paulo, verifying the association between the patterns of use and the variables of the sample.

**Materials and methods:** Cross-sectional study carried out in a primary health care Unit in the downtown of São Paulo. Data were collected from July 2017 to February 2018. Alcohol Use Disorders Identification Test -AUDIT was used to identify the pattern of alcohol use. From the database, a descriptive analysis was performed and to investigate the association we used the Kendall correlation test and the Kruskal–Wallis test.

**Results:** The study sample consisted of 561 women, with a mean age of 43.27 years, The majority of participants were heterosexual (n = 529, 94.8%), browns (n = 244, 43.9%), single (n = 210, 43.9%), completed high school (n = 197, 35.1%), smokers (n = 98, 17.5%) and illicit drug users (n = 20, 3.6%). There was an association between higher alcohol use patterns with being homosexual (p < 0.00), being single (p < 0.00), have no religion (p < 0.00) and being a student (p = 0.02).

**Conclusions:** Women attended in primary health care services should be screened with the aim of identifying their pattern of alcohol consumption and should be especially attentive to those who are homosexual, single, students and who do not have any religion.

## A24 Novel approaches to measuring and addressing the common challenges of referral to treatment and long-term sustainability in SBIRT

### Stacy A. Sterling^1^, Wendy Lu^1^, Constance M. Weisner^1^, Amy S. Leibowitz^1^, Derek Satre^2^

#### ^1^Division of Research, Kaiser Permanente Northern California, Oakland, CA, USA; ^2^Department of Psychiatry, University of California, San Francisco, CA, United States

##### **Correspondence:** Stacy A. Sterling (stacy.a.sterling@kp.org)

*Addiction Science & Clinical Practice* 2018, **13(Suppl 1)**: A24

**Background:** Alcohol screening, brief intervention, and referral to treatment (SBIRT) is increasingly being implemented in health systems, but numerous challenges persist which can impact efficacy, effectiveness, and sustainability. We focus on challenges to SBIRT implementation and long-term sustainability, and illustrate novel approaches being used to study them, in the context of a large-scale alcohol SBIRT initiative in Kaiser Permanente Northern California (KPNC), a large, integrated healthcare system.

**Materials and methods:** The implementation of systematic alcohol SBIRT in adult primary care in KPNC was informed by an NIAAA-funded cluster-randomized implementation trial (ADVISe) of different modalities of SBIRT delivery, and involves: EHR-embedded, evidence-based alcohol screening instruments and clinical decision support tools; medical assistants (MA) and primary care providers (PCPs) trained in screening, brief intervention (BI) and referral techniques; and curricula and workflows developed for MA and PCP training, skills reinforcement, and troubleshooting. Structures were created for measuring, communicating and reinforcing provider- and facility-level EHR date on screening and BI performance.

**Results:** The initiative conducts an average of 145,000 alcohol screenings each month (on 86% of adult primary care patients), and 9500 BIs. Since its adoption in July, 2013, there have been 7.7 million alcohol screenings conducted, and 468,507 BIs delivered. However, there is significant variation in implementation outcomes (e.g., BI rates varying from 32% to 83% across medical centers, and from 0% to 100% among PCPs) across medical centers, clinics and between primary care providers (PCPs), and little is known about the factors causing this variability, and those contributing to long-term sustainability of SBIRT. We describe a new approach to studying factors associated with successful SBIRT implementation and long-term SBIRT sustainability. Informed by the PRISM theoretical framework, this mixed-method study uses EHR data, PCP surveys, qualitative key informant interviews and patient interviews to examine intervention-, organizational-, provider-, environmental- and recipient-level factors, including assessments of patient and provider experiences of BI quality and fidelity.

**Conclusions:** We discuss the challenges to successful implementation of universal SBIRT in health system settings, and make recommendations for approaches to evaluating factors associated with its long-term sustainability.

## A25 Incorporating HIV discussions into brief interventions: lessons learned from SBIRT research in primary care settings

### Shannon G. Mitchell, Robert P. Schwartz, Jan Gryczynski

#### Friends Research Institute, Baltimore, MD, USA

##### **Correspondence:** Shannon G. Mitchell (smitchell@friendsresearch.org)

*Addiction Science & Clinical Practice* 2018, **13(Suppl 1)**: A25

**Background:** The implementation of screening and brief intervention within primary care settings creates opportunities to also discuss HIV-related risk behaviors, however, little has been documented with respect to bridging such conversations within the parameters of a primary care visit. The proposed presentation will examine the integration of HIV-discussions across three different SBIRT studies, which included adult patients in two rural Federally Qualified Health Centers (FQHCs), adolescent patients in an urban FQHC, and adolescent patients in two urban school-based health centers.

**Materials and methods:** Utilizing a mixed-methods approach, we analyzed data across the three studies. Descriptive statistics were used to examine quantitative data concerning patients’ reported alcohol and substance use. Semi-structured qualitative interviews were conducted with eight primary care providers to understand factors influencing the integration of HIV-discussions with alcohol and drug-related brief interventions (BIs). Qualitative data were analyzed using Atlas.ti to capture emergent and anticipated themes.

**Results:** Marijuana was the most frequently used substance reported, followed by alcohol use in the study samples. Reported use of any other drugs was extremely low in these primary care samples. Qualitative interviews with primary care providers revealed several factors influencing whether or not they discussed HIV-related risk behaviors during BIs, including: the substance used, the presenting problem, the patient’s medical history, and the length of the medical appointment. Providers mentioned greater comfort levels discussing sexual risk behaviors than drug-related behaviors, and focusing on the same patient-driven decisional points when balancing harm reduction and abstinence issues. The provider’s comfort with motivational interviewing techniques seemed to indicate a willingness to use the techniques with topics beyond alcohol and substance use.

**Conclusions:** While HIV-related risk behaviors may be low in primary care settings, opportunities are still present to expand the impact of drug or alcohol-related brief interventions by bridging these two related health topics.

## A26 Adolescent SBIRT in pediatric primary care: Healthcare utilization outcomes from a randomized trial in an integrated healthcare system

### Stacy A. Sterling^1^, Sujaya Parthasarathy^1^, Ashley Jones^1^, Derek Satre^2^, Constance Weisner^1,2^, Andrea Kline-Simon^1^

#### ^1^Division of Research, Kaiser Permanente Northern California, Oakland, CA, USA; ^2^Department of Psychiatry, University of California San Francisco, San Francisco, CA, USA

##### **Correspondence:** Stacy A. Sterling (stacy.a.sterling@kp.org)

*Addiction Science & Clinical Practice* 2018, **13(Suppl 1)**: A26

**Background:** Little is known about the effects of Screening, brief intervention, and referral to treatment (SBIRT) on subsequent healthcare utilization among adolescents. We describe health care utilization findings from a trial of SBIRT in pediatric primary care.

**Materials and methods:** We randomized clinic pediatricians (n = 52) to three study arms: (1) pediatrician-only, in which they were trained to deliver SBIRT; (2) embedded-BHC arm, in which they could refer patients to a behavioral health clinician (BHC); and (3) Usual Care (UC). We used electronic health record (EHR) data to obtain all inpatient and outpatient utilization (emergency department (ED), primary care (PC), Psychiatry and Chemical Dependency) during the 3 years following the index visit, across the three arms. We used logistic regression to examine any ER or inpatient use and negative binomial distribution for PC, psychiatry, Chemical Dependency and total outpatient visit count. The exponent of the estimated coefficient represents the odds ratio or rate ratio, with a value < 1 indicating less likelihood of a visit (for logistic) or fewer visits (for negative binomial).

**Results:** There were 1871 eligible patients. At 1-year post-index, there were no differences in PC or inpatient utilization between the arms. Compared to UC, adolescents in the BHC arm had less likelihood of ED use (adjusted odds ratio [aOR] = 0.60, 95%CI = 0.34, 1.05), and were likely to have fewer Psychiatry visits (adjusted rate ratio [aRR] = 0.73, 95%CI = 0.52,1.02), or any outpatient utilization (aRR = 0.67, 95% CI = 0.51,0.87). Adolescents in the PCP arm also had less likelihood of ED use than those in UC (aOR = 0.76, 95% CI = 0.59,0.98). At 3-years post-index, there were no differences in the likelihood of PC, inpatient or ED utilization across the arms. However, compared to those in the UC arm, adolescents in the BHC and PCP arms had fewer Psychiatry visits (aRR for BHC: = 0.56, 95% CI = 0.41,0.76; aRR for PCP: = 0.73, 95% CI = 0.53,0.99), or any outpatient services (aRR for BHC = 0.76, 95% CI = 0.64, 0.91; aRR for PCP = 0.78, 95% CI = 0.65, 0.93).

**Conclusions:** Adolescents in both intervention arms were less likely to use several types of healthcare, including ED services. SBIRT, delivered as part of pediatric primary care, may obviate the need for potentially more costly specialty and emergency care.

## A27 Family members of individuals suffering from addiction: A target group for brief interventions?

### Gallus Bischof^1^, Christian Meyer^2^, Anil Batra^3^, Johannes Berndt^1^, Bettina Besser^1^, Anja Bischof^1^, Sandra Eck^3^, Kristian Krause^2^, Anne Moehring^2^, Hans-Jürgen Rumpf^2^

#### ^1^Department of Psychiatry and Psychotherapy, University of Luebeck, Luebeck, Germany; ^2^Institute of Social Medicine and Prevention, University Medicine of Greifswald, Greifswald, Germany; ^3^Department of Psychiatry and Psychotherapy, Universiy Medical Center Tuebingen, Tuebingen, Germany

##### **Correspondence:** Gallus Bischof (gallus.bischof@uksh.de)

*Addiction Science & Clinical Practice* 2018, **13(Suppl 1)**: A27

**Background:** Relatives of individuals with addictive disorders (RIAD) show elevated health-related morbidity but rarely seek help in the addiction treatment system. The aim of the present study is to estimate the prevalence of the problem and the level of impairment in RIAD identified via pro-active screening in primary care settings.

**Materials and methods:** As a part of a health screening, patients aged 18–64 years. from general practices and general hospitals (N = 2273) were asked if they had a relative with a present or remitted addictive disorder (tobacco use disorder excluded). Relationship status and type of addiction were specified. In addition, depressive mood and health behaviour of patients were assessed. Patients without addicted relative were compared to patients with current or remitted addicted relatives, respectively.

**Results:** In the whole sample, 12.7% (95% CI 11.4–14.0) of all respondents mentioned to have a relative with a present addictive disorder, and another 6.5% (95% CI 5.6–7.4) reported to have a relative with a remitted addictive disorder. Prevalence rates were significantly elevated in general hospital patients. When controlling for sociodemographic variables and health-related behaviours, relatives revealed elevated depression scores compared to controls.

**Conclusions:** Relatives of individuals with addictive disorders are a vulnerable population and highly prevalent among patients in primary care. Evidence-based treatment including brief interventions exist and might have a public-health impact.

## A28 Documented brief intervention associated with reduced linkage to treatment in a national sample of patients with unhealthy alcohol use with and without alcohol use disorders

### Emily C. Williams^1,2^, Joseph E. Glass^3^, Madeline C. Frost^1^, Katharine A. Bradley^3^

#### ^1^Department of Health Services, University of Washington, Seattle, WA, USA; ^2^Veterans Affairs Puget Sound Health Care System, Health Services Research & Development, Seattle, WA, USA; ^3^Kaiser Permanente Washington Health Research Institute, Seattle, WA, USA

##### **Correspondence:** Emily C. Williams (Emily.Williams3@va.gov)

*Addiction Science & Clinical Practice* 2018, **13(Suppl 1)**: A28

**Background:** Alcohol screening, brief intervention (BI), and referral to treatment initiatives are often considered stepped care, such that BI will help link patients to treatment. A meta-analysis of BI trials found no evidence that BIs increase treatment receipt, but this has not been evaluated among patients receiving BI as part of routine care. We evaluate this question in a national healthcare system in which BI provision is encouraged annually by performance measurement and supported by electronic clinical decision support.

**Materials and methods:** Secondary national clinical and administrative data from the U.S. Veterans Health Administration (VA) were used to identify all positive alcohol screens (AUDIT-C score ≥ 5) documented nationally (10/01/09–5/30/13). Regression models were used to estimate the prevalence of receiving specialty addictions treatment within 365 days after screening positive for patients with and without documented BI (advice to reduce or abstain ≤ 14 days of positive screen), in patients with and without diagnosed alcohol use disorder (AUD; based on ICD codes). Models were adjusted for demographics and mental health and substance use conditions and clustered on patient. Sensitivity analyses additionally adjusted for prior treatment receipt were conducted within a limited sample in which documentation of prior treatment was available.

**Results:** Among 830,825 VA outpatients with positive screens for unhealthy alcohol use (1172,606 positive screens), 36% had diagnosed AUD, 74% received BI, and 11% received addictions treatment. Documented BI was associated with a decreased likelihood of receiving addictions treatment (Adjusted IRR 0.86, 95% CI 0.84–0.88). For patients without documented AUD, the prevalence of addictions treatment was lower for those with BI (4.0%; 95% CI 3.9–4.1) than those without (4.7%; 4.5–4.8). Similarly, among patients with AUD, those with BI documented had a lower prevalence of addictions treatment than those without [19.7% (19.5–19.9) and 16.4% (16.3–16.5)]. Results were similar in sensitivity analyses.

**Conclusions:** In this national sample of patients with unhealthy alcohol use, documentation of BI was associated with a decreased likelihood of receiving specialty addictions treatment, for patients with and without AUD. These findings do not support the notion that BI increases linkage to treatment.

## A29 Brief intervention for alcohol use in pregnant women: evidence of newborns health indicators in Argentina

### Aldana Lichtenberger, Karina N. Conde, Raquel I. Peltzer, Paula Gimenez, Mariana Cremonte

#### Research Group on Psychoactive substances and injuries- Institute of Basic Psychology, Applied and Development of Psychological Technology (IPSIBAT)- National Scientific and Technical Research Council (CONICET)- National University at Mar del Plata, Mar del Plata, Argentina

##### **Correspondence:** Aldana Lichtenberger (aldanalich@hotmail.com)

*Addiction Science & Clinical Practice* 2018, **13(Suppl 1)**: A29

**Background**: Alcohol is a teratogen that reaches the fetus through the placenta and increases the risk of fetal death, spontaneous abortions, under birth weight, premature birth, low gestational age, and fetal alcohol spectrum disorders. These consequences are 100% preventable if no alcohol is consumed during pregnancy. The aim of this work was to evaluate the efficacy of a brief intervention (BI) taking newborns health indicators as an objective outcome measure.

**Materials and methods**: We screened 503 pregnant women up to 26 weeks of gestation attending Public Health Centers of the Municipality of General Pueyrredón, Argentina, during 2016. We performed a probabilistic sampling, with random assignment between two groups: alcohol screening and BI or alcohol screening and brief advice (BA). After childbirth, we obtained health indicators from the newborns: birth weight in kilograms, gestational age at birth in weeks, and APGAR score in a range from 1 to 10 (BI group = n = 77; BA group = n = 72). In addition, we included a third control group (EC) of newborns whose mothers did not participate in the alcohol screening groups (n = 150). We compared newborns health indicators from BI, BA and EC groups with each other using the Wilcoxon Ranke Test and the Cliff´s Delta analysis as a measure of effect size. Analyses were performed with the R Project for Statistical Computing, version 3.4.1.

**Results**: We registered statistically significant differences in the birth weight (p < .05) and gestational age at birth (p < .001) between BI and BA groups compared with the third control group (EC). No statistically significant differences were found in the values of the APGAR score. The effect size of the differences found was modest. We did not find statistically significant differences in any of the three indicators (birth weight, APGAR and gestational age at birth) among BI and BA groups.

**Conclusions**: Our results suggest that alcohol screening and brief intervention or brief advice among pregnant women significantly reduce the newborns’ risk of suffering some of the consequences of prenatal alcohol exposure.

## A30 Patients’ acceptance of alcohol screening and brief interventions in general hospitals

### Torgeir G. Lid^1,2,3^, Hege Tvedt^3^, B. Nathalie Idsøe^1^, Inger B. Hustvedt^1^, Sverre Nesvåg^1,3^

#### ^1^Center for Alcohol and Drug Research, Stavanger University Hospital, Regional Health Trust of Western Norway, Stavanger, Norway; ^2^Research Unit for General Practice, Uni Health, Uni Research, Bergen, Norway; ^3^Faculty of Health Sciences, University of Stavanger, Stavanger, Norway

##### **Correspondence:** Torgeir G. Lid (torgeir.gilje.lid@sus.no)

*Addiction Science & Clinical Practice* 2018, **13(Suppl 1)**: A30

**Background:** Alcohol consumption in Norway increased by 40% in two decades from the early 90s. Even though the general public is well acquainted with the concepts of addiction and abuse, the awareness of alcohol as a potentially relevant factor for a whole array of clinical problems, without any signs of addiction or abuse, have been scarce. Since 2013, all general hospitals in Norway are obliged to identify and to intervene with risky or harmful alcohol consumption, but such strategies are still lacking in many hospitals. Stavanger University Hospital has had an alcohol liaison team since 2008. In this study, we wanted to explore how patients without prior alcohol or drug use disorder (AUD/SUD) experienced the interventions by the alcohol liaison team.

**Materials and methods:** Patients admitted to Stavanger University Hospital without previous alcohol or drug use disorder and without previous brief alcohol interventions (BAI) where invited, after a brief alcohol intervention. Patients who accepted went through a telephone interview one week after the hospital stay.

**Results:** In the study period, 182 patients without previous AUD/SUD or previous BAI received a BAI from the alcohol liaison team. Of these, 91 patients accepted to take part in the study, and 58 patients completed the interview. A large majority of the patients said that they had been informed well or very well about the reasons for the intervention, and that they themselves understood well or very well the reasons for the intervention. Almost all patients found the intervention relevant for their own health, but less than half of the patients believed that alcohol would be addressed when seeing their GP later.

**Conclusions:** This study indicates that patients without previous diagnoses or interventions are accepting BAI when healthcare personnel finds it relevant. Fewer patients expect alcohol to be addressed when seeing their GP later. This may be because they do not find it serious enough or they believe they will solve the problem, or because of a lack of trust in the relationship with their GP or the GP’s abilities. Further studies should explore patients’ perspectives on collaboration between primary care and hospitals.

## A31 Effectiveness of a web-based screening and brief intervention with weekly text-message-initiated individualized prompts for reducing risky alcohol use among teenagers

### Silke Diestelkamp^1^, Lutz Wartberg^1,2^, Rainer Thomasius^1^

#### ^1^German Center for Addiction Research in Childhood and Adolescence, Center for Psychosocial Medicine, University Medical Center Hamburg-Eppendorf, Hamburg, Germany; ^2^Medical School Hamburg, Hamburg, Germany

##### **Correspondence:** Silke Diestelkamp (s.diestelkamp@uke.de)

*Addiction Science & Clinical Practice* 2018, **13(Suppl 1)**: A31

**Background**: While at-risk drinking is prevalent among 16% of male 11- to 17-year-olds (12% among females) in Germany, prevention and early intervention only reaches a small percentage of youth. Evidence supports the effectiveness of electronic alcohol interventions in reducing alcohol consumption and related harms in populations of young drinkers, especially when participants are contacted multiple times over the duration of an intervention. This trial aims to evaluate the effectiveness and cost-effectiveness of a single session brief motivational web-based intervention (Pro-WISE) plus weekly text-message-initiated individualized prompts (TIPs) delivered over a period of 3 months in reducing alcohol consumption and alcohol-related harm.

**Materials and methods**: The Pro-WISE-TIP trial is part of the multicenter Pro-HEAD study which aims at the prevention of mental health problems (alcohol misuse, depression, eating disorders) in 12- to 17-year-olds. In a four-arm, randomized controlled design the following groups will be compared in the Pro-WISE-TIP trial: (A) web-based intervention plus text-message-initiated individualized prompts for 12 weeks, (B) web-based intervention plus text-message-initiated assessment of alcohol consumption for 12 weeks, (C) web-based intervention only, (D) psychoeducation. TIPs are tailored to individual differences in drinking motives, age and gender and are designed to reach youth in the contexts of their everyday lives by providing individualized feedback on drinking intentions, actual drinking and succession in achieving goals for low-risk drinking or abstinence. In the Pro-HEAD study, a target sample of N = 15.000 will be recruited in schools. Those with a positive screening (≥ 2) for at-risk alcohol use in the CRAFFT-d will be included in the Pro-WISE-TIP trial (target n = 1076). Primary outcome is alcohol use in the past 30 days 9 months after enrollment. Secondary outcomes are alcohol-related problems, co-occurring substance use, further health service utilization, mental health problems and health-related quality of life. Study participants will be followed up at 3, 6 and 9 months in the Pro-WISE-TIP trial and at 1 and 2 years in the Pro-HEAD study.

**Results and conclusions:** The Pro-WISE-TIP intervention, if effective, can be used as a stand-alone youth-specific brief alcohol intervention or as an add-on to future school-based or community-based alcohol prevention programs.

## A32 Evaluation of a cognitive behavioral intervention in cartoon format for alcohol abuse designed for the working population. Results of a pilot test

### Nora A. Martínez Vélez, Shoshana Berenzon Gorn, María Elena Medina-Mora, Marcela A Tiburcio Sainz

#### Departamento de Ciencias Sociales en Salud, División de Investigaciones Epidemiológicas y Psicosocialesde, Instituto Nacional de Psiquiatría Ramón de la Fuente Muñiz, Ciudad de México, México

##### **Correspondence:** Nora A. Martínez Vélez (martinve@imp.edu.mx)

*Addiction Science & Clinical Practice* 2018, **13(Suppl 1)**: A32

**Background**: The implementation of preventive and treatment actions to reduce alcohol consumption in the work environment can be complex due to the lack of interest on the part of employers. Moreover, employees do not always have time during working hours to participate in these activities. It is therefore important to adapt preventive interventions to simple, practical and efficient modalities. Objective: Evaluate the efficiency of a behavioral cognitive intervention in cartoon format for alcohol abuse designed for the working population.

**Materials and methods:** A randomized clinical pilot test was performed with 42 workers ages 19–50 from three different firms. Respondents were arbitrarily assigned to one of three conditions: (1) Cognitive-behavioral intervention in cartoon format with weekly accompaniment by a monitor (C + M), (2) Cognitive-behavioral intervention in cartoon format (C) and (3) Brief advice through a brochure (BA). Alcohol consumption and associated work problems were assessed through AUDIT before and after the intervention.

**Results:** The percentage of people with dangerous consumption fell from 10% to 0% in the C + M group, from 20% to 16.7% in the cartoon group and from 40% to 28.6% in the BA group. The number of absences and late arrivals associated with consumption also fell. A total of 20% completed the cartoon activities, 50% felt relaxed after doing the activities and 40% felt that what they had learned could help in other aspects of their life.

**Conclusions:** The cartoon format is a short intervention alternative to assist the working population in the workplace who lack the time and resources to seek another type of treatment. However, a randomized clinical trial to confirm the effectiveness of this tool is required.

## A33 Evaluation of a brief intervention protocol for reducing alcohol consumption by pregnant women attending the Brazilian Unified Health System

### Erikson F. Furtado^1^, Poliana P. Aliane^2^, Joseane de Souza^3^, Larissa E. Horta^4^

#### ^1^Child and Adolescent Psychiatry Section, Department of Neurosciences and Behavior, School of Medicine of Ribeirao Preto, University of Sao Paulo, Sao Paulo, Brazil; ^2^Health Personal Continuing Education of the City Health Department, Municipality of Araraquara, Brazil; ^3^Guairacá Faculty, Guarapuava, Brazil; ^4^School of Nursing of Ribeirao Preto, University of Sao Paulo, Brazil

##### **Correspondence:** Erikson F. Furtado (efurtado@fmrp.usp.br)

*Addiction Science & Clinical Practice* 2018, **13(Suppl 1)**: A33

**Background:** In Brazil, different studies indicated about 50% of women reporting drinking alcohol anytime during pregnancy. When considering the level of risk, with T-ACE screening, there is 20% pregnant women at risk. This study aimed to evaluate a newly developed brief intervention protocol for pregnant women, based on one single face-to-face one-hour intervention in the first weeks of gestation against another preventive strategy based on the reading of a self-assessment and orientation brochure, likewise specially developed for this purpose and for this research.

**Materials and methods:** The study design was a blind, comparative and prospective clinical trial. After the screening of 82 T-ACE positive adult pregnant women in the first gestational trimester, they were distributed equally in two groups, with a random distribution of participants, (brief intervention group and brochure group). Currently abstinent pregnant women, alcohol or drug dependent women (with the exception of tobacco) were excluded from the sample, as well as those presenting any psychiatric symptoms or treatment. A follow-up and outcome assessment was performed in the third gestational trimester with 73 study subjects (11% attrition rate). Alcohol variables include AUDIT scores (total and by item), report of recent drinking pattern, in terms of frequency and quantity (by standard drink).

**Results:** The intragroup evaluation showed a statistically significant reduction for the total sample (Wilcoxon, Z = − 5.05, p < 0.001), and for both, the brochure group (Wilcoxon, Z = − 2, 46, p < 0.01) and the Brief Intervention group (Wilcoxon, Z = − 4.59, p < 0.001). The effect size of the interventions (brochure and brief intervention) was calculated through Cohen’s r. For the brochure group, the r value was 0.42 indicating that this explained 17.6% of the variance (mean effect following the Cohen classification). For the brief intervention group, the r value was 0.74, indicating that this intervention explained 54.8% of the variance (large effect following the Cohen classification).

**Conclusions:** Brochure and BI interview, showed a reduction in alcohol consumption. A small increase in the proportion of abstinent pregnant women was found among the BI group. Unfortunately, none of the interventions have been found to be effective in promoting total abstinence in this high-risk group.

## A34 Identification of alcohol problem use and alcohol use disorder in adults primary care patients: TAPS compared with AUDIT-C and ASSIST

### Angeline Adam^1^, Robert P. Schwartz^2^, Li-Tzy Wu^3^, Geetha Subramaniam^4^, Gaurav Sharma^5^, Eugene Laska^1^, Jennifer McNeely^1^

#### ^1^Department of Population Health, New York University School of Medicine, New York, NY, USA; ^2^Friends Research Institute, Inc., Baltimore, MD, USA; ^3^Department of Psychiatry and Behavioral Sciences, Department of Medicine and Duke Clinical Research Institute, Duke University Medical Center, Durham, NC, USA; ^4^Center for the Clinical Trials Network, National Institute on Drug Abuse, National Institutes of Health, Bethesda, MD, USA; ^5^The EMMES Corporation, Rockville, MD, USA

##### **Correspondence:** Angeline Adam (angeline.adam@nyumc.org)

*Addiction Science & Clinical Practice* 2018, **13(Suppl 1)**: A34

**Background:** The TAPS is a brief screening instrument to identify tobacco, alcohol, and drug use in primary care patients. This secondary analysis compares the TAPS to the Alcohol Use Disorders Identification Test (AUDIT-C) and Alcohol, Smoking and Substance Involvement Screening Test (ASSIST) for identifying alcohol problem use and alcohol use disorder (AUD).

**Materials and methods:** 2000 patients in diverse U.S. primary care sites completed interviewer-administered and computer self-administered versions of the TAPS, followed by additional measures: 1-ASSIST, 2-AUDIT-C, and 3-modified CIDI substance abuse module (CIDI). The sensitivity, specificity, and area under the curve (AUC) of the three instruments were evaluated in men and women using the CIDI as the reference standard for problem use (CIDI cut-off ≥ 1) and AUD (CIDI cut-off ≥ 2) according to the DSM-5.

**Results:** The performance of the self-administered and interviewer-administered TAPS was similar, and results for the self-administered TAPS are presented. Problem use: Among women (n = 1124), the TAPS (cut-off ≥ 1) had sensitivity 0.76, specificity 0.78, and AUC 0.77. ASSIST (cut-off ≥ 6) had sensitivity 0.80, specificity 0.81, AUC 0.81. AUDIT-C (cut-off ≥ 2) had higher sensitivity (0.89), and specificity 0.78, AUC 0.83. Results were similar for men (n = 874): TAPS (cut-off ≥ 1) had sensitivity 0.76, specificity 0.76, AUC 0.76. ASSIST (cut-off ≥ 5) had sensitivity 0.83, specificity 0.71, AUC 0.77. AUDIT-C (cut-off ≥ 3) had sensitivity 0.84, specificity 0.82, AUC 0.83.

AUD: Among women, TAPS (cut-off ≥ 2) had sensitivity 0.73, specificity 0.85, AUC 0.79. ASSIST (cut-off ≥ 7) had sensitivity 0.79, specificity 0.87, AUC 0.83. AUDIT-C (cut-off ≥ 3) had higher sensitivity (0.83), with specificity 0.83, AUC 0.83. Similarly among men, TAPS (cut-off ≥ 2) had sensitivity 0.75, specificity 0.84, AUC 0.79 and performed similarly to the ASSIST (cut off ≥ 10), which had sensitivity 0.73, specificity 0.91, AUC 0.82. AUDIT-C (cut-off ≥ 4) performed best, with sensitivity 0.81, specificity 0.84, AUC 0.83.

**Conclusions:** At the recommended cut-offs, all instruments demonstrated adequate performance for alcohol screening in primary care patients, and AUDIT-C had the highest sensitivity and specificity. Among the instruments that integrate alcohol with other substances screening, the self-administered TAPS Tool performed similarly to the longer ASSIST and may be considered as an alternative.

## A35 Development of a mobile-based brief intervention treatment for hazardous drinkers in Goa-India

### Sheina P. Costa^1^, Devika Gupta^1^, Urvita Bhatia^1^, Richard Velleman^2^, Abhijit Nadkarni^1^

#### ^1^Addictions Research Group, Sangath-Goa, Porvorim, India; ^2^Department of Psychology, University of Bath, Bath, UK

##### **Correspondence:** Sheina P. Costa (sheina.costa@sangath.in)

*Addiction Science & Clinical Practice* 2018, **13(Suppl 1)**: A35

**Background**: Despite the high public health burden of hazardous drinking in India, policy focuses predominantly on alcohol dependence which affects a much smaller proportion of Indians. A growing body of evidence demonstrates the effectiveness of Brief Interventions (BIs) in reducing alcohol consumption in Hazardous Drinkers (HDs). Our study aims to understand the acceptability and feasibility of using mobile-based technology such as Short Message Service (SMS) and/or Interactive Voice Response (IVR) calls to deliver a contextually appropriate brief intervention for adult HDs and further inform the development of such an intervention.

**Materials and methods**: A relevant and recent systematic review was identified, and data were systematically extracted for the purpose of the study. Ten in-depth qualitative interviews were conducted with national experts to explore their perceptions of the acceptability and feasibility of developing and delivering a mobile-based BI treatment in a low resource setting. Additionally, 150 intended recipients of the BI package across primary health care centres, educational institutions, and workplaces were also screened for HD using the Alcohol Use Disorder Identification Test (AUDIT). Identified HDs were then interviewed to seek their perspectives on the content and delivery platforms to be considered before developing a mobile-based BI. A thematic analysis of interview transcripts was conducted using Nvivo version 11. Lastly, the information collected through the systematic review and qualitative interviews was triangulated to inform the next step of the intervention process.

**Results:** Study findings revealed that majority of the HDs expressed feeling comfortable using a mobile phone, specifically through SMS to seek treatment for their condition. Most of the participants opted to be contacted weekly and preferably on weekends. Lastly, both experts and intended recipients felt that information on ill effects of alcohol, managing cravings and tempting situations and helpful resources in case of emergencies would be important to include as part of the BI.

**Conclusions:** Preliminary findings suggest that a mobile-based BI will be acceptable to the intended recipients and feasible to deliver.

## A36 Alcohol consumption by older people in Brazil: a systematic review of population-based studies

### Camila Chagas^1^, Danusa A Machado^1^, Leonardo B. Martins^1,2,3^, Davi Opaleye^1^, Tatiani Piedade^1^, Tassiane C. S. Paula^1^, Cleusa P. Ferri^4^

#### ^1^Department of Psychobiology, Federal University of São Paulo, São Paulo, Brazil; ^2^Inter Psi: Laboratório de Psicologia Anomalística and Processos, Institute of Psychology, Universidade de São Paulo, São Paulo, Brazil; ^3^Laboratório de Psicologia Social da Religião, Universidade de São Paulo, São Paulo, Brazil; ^4^Health Technology Assessment Unit, Institute of Education and Health Sciences, Hospital Alemão Oswaldo Cruz, São Paulo, Brazil

##### **Correspondence:** Camila Chagas (psicologia.chagas@gmail.com)

*Addiction Science & Clinical Practice* 2018, **13(Suppl 1)**: A36

**Background**: With the rapid ageing process happening in low and middle-income countries like Brazil, it is important to know the pattern of alcohol consumption by older people and the factors associated with this behaviour.

**Materials and methods**: For this systematic review a search in MEDLINE, EMBASE and LILACS for studies published between 1980 and 2017 was conducted to identify population-based studies written in English, Portuguese or Spanish, on the prevalence of different patterns of alcohol consumption among Brazilians aged 60 and over.

**Results:** Eight studies were identified, all of them were published after 2000 (six in 2000’s and two in 2010’s). The sample size varied from 123 to 2143. Only two studies reported data on binge drinking, one conducted in a representative sample of the country defined as 3 drinks in one occasion and found a prevalence of 10.3%, while another investigated two cities defining binge as 5 drinks on one occasion and found a prevalence of 27.1% (Belo Horizonte) and 13.7% (Bambuí). Heavy drinking was estimated in four studies using different definitions and prevalence varied from 2.9 to 7.3%. Three studies provided estimates for alcohol dependence. Only one study used the DSM-IV diagnosis criteria and found a prevalence of alcoholism of 3.8%, whilethe other two used screening tools and found higher prevalence, 8.2% and 9.2. Male gender and younger ageweres found to be associatedwitho most patterns of alcohol consumption. High education was associatedwitho binge and heavy drinking, while low education and low socioeconomic status to alcohol dependence.Other factorsr such as, being separated/divorced, disability, smoking and falls were reported to be associatedwitho one pattern of alcohol drinking.

**Conclusions:** These findings show that in Brazil, the problems related to alcohol use by the elderly remain relatively unknown, since there are few studies on this subject. Some instruments used in the methodology of the studies may not be suitable for the population over 60 years. In addition, the divergence in the methodology adopted may explain the variations observed in the prevalence of alcohol consumption and the factors associatedwitho its use.

## A37 Brief intervention program for adolescents abusing alcohol and other drugs (PIBA): history and challenges and its implementation in school scenarios in Mexico

### Kalina I. Martínez Martínez^1,2^, Francisco J. Pedroza Cabrera^1^, Hugo E. Reyes Huerta^1^

#### ^1^Departamento de psicología, Universidad Autónoma de Aguascalientes, Aguascalientes, México; ^2^Facultad de psicología, Universidad Nacional Autónoma de México, Ciudad de México, México

##### **Correspondence:** Kalina I. Martínez Martínez (kalinamartinez@hotmail.com)

*Addiction Science & Clinical Practice* 2018, **13(Suppl 1)**: A37

PIBA has proved to have a positive impact on the reduction of adolescents’ consumption patterns when comparing baseline data with treatment and follow-up results. Likewise, there is an increase in the self-efficacy of teens to face situations of consumption, and also in the reduction of the number of problems associated with it. These results have been replicated in different samples of women and men living in urban or rural areas.

On the other hand, the effectiveness of PIBA also seems to be related with the adaptations that have been made of the materials, instruments and sessions; the adaptation of training online, the introduction ofand induction session, and the adaptation of materials forthe rural population.

In addition, a randomized clinical trial was conducted, where a control group is included and the comparison between two interventions. Also, it has been compared against the brief advice (CB); and shows both treatments significantly decrease the consumption and level of risk compared with the control group, but PIBA showsan even greater reduction in the consumption rates. So the foregoing, allowslocatinge PIBA as a treatment in constant evaluation in Mexico.

In the present, we continue to investigate new areas of interest in relation to the program and keep working to achieve a successful transfer to the users of this type of brief interventions.

## A38 The impact of a lay health counsellor delivered psychological treatment for harmful drinking in primary care: A qualitative study nested in the PREMIUM trial in Goa, India

### Urvita Bhatia^1^, Sachin Shinde^1^, Abhijit Nadkarni^1,2^, Richard Velleman^1,3^, Vikram Patel^1,4^

#### ^1^Addictions Research Group, Sangath, Porvorim, India; ^2^King’s College London, London, UK; ^3^University of Bath, Bath, UK; ^4^Harvard Medical School, Boston, MA, USA

##### **Correspondence:** Urvita Bhatia (urvita.bhatia@sangath.in)

*Addiction Science & Clinical Practice* 2018, **13(Suppl 1)**: A38

**Background:** Alcohol consumption is a major public health concern in India because of an increase in availability of alcohol, rapid changes in the patterns of alcohol use (frequent and heavy drinking), and alcohol-related problems. Alcohol Use Disorders (AUDs) have been accorded a low priority in India and evidence-based brief interventions have limited access because of low help-seeking rates, lack of human resources, and limited contextual applicability of interventions which are developed in a different setting. The PREMIUM trial demonstrated the effectiveness and cost-effectiveness of Counselling for Alcohol Problems (CAP), a brief psychological treatment delivered by lay counsellors to patients with harmful drinking attending primary health-care settings. The qualitative sub-study explored the experiences of receiving CAP and its perceived impact.

**Materials and methods**: Semi-structured qualitative interviews with 51 trial participants, 33 were from the CAP arm, and 18 from the Enhanced Usual Care (EUC) arm. We used thematic analysis to analyse the data.

**Results:** The average age was 45 years, withthe majority of the participants being married (75%) and employed (78%), with the most common occupation being daily wage labour, domestic work, and farming (68%). Participants explained that the most preferred change in drinking patterns wasa reduction in drinking. Participants highlighted differences in drinking patterns before and after treatment in terms ofthe quantity of alcohol consumed, the frequency of alcohol consumed, amount of money spent on buying alcohol, situations where alcohol is consumed, etc. Participants highlighted positive changes in lifestyle, sleep and appetite, physical and mental health, relationships, and work functioning. The counsellor’s advice which was perceived as helpful encompassed understanding drinking patterns, its impact, strategies to change drinking behaviours, and choosing a preferred goal. The specific strategies cited were maintaining personal resolve, focussing on the negative impact of drinking, controlling urges, and distracting oneself. Participants reported better engagement with CAP because of certain qualities of the counsellor, who was seen to be understanding, friendly, non-forceful and non-judgemental.

**Conclusions:** Contextualised brief psychological treatments for harmful drinking are acceptable and effective when delivered by non-specialist health workers in routine health-care. Such treatments need to be scaled up to address the high-unmet need associated with AUDs.

## A39 Evaluation of the implementation process of the early detection, brief intervention and referral to treatment (SBIRT) in Chile

### Fernando Poblete, María Soledad Zuzulich, Nicolas Barticevic Lantadilla, Magdalena Galarce, Constanza Vargas, Aracelly Godoy, Bárbara Bustos, Luis Villarroel

#### Pontificia Universidad Católica de Chile, Santiago de Chile, Chile

##### **Correspondence:** Fernando Poblete (fcpoblete@gmail.com)

*Addiction Science & Clinical Practice* 2018, **13(Suppl 1)**: A39

**Background:** In 2011, a pilot implementation program was launched to assess the effectiveness of the SBIRT model as barriers and facilitators in the implementation process in the community, including Primary Health Care (PHC) and Police Stations. Analyze the process of implementing a model of a community system for early detection, brief intervention, and referral to treatment (SBIRT) in people with alcohol and drug risk consumption.

**Materials and methods:** A multiple case study was carried out, applying mixed methods. The surveyed population was 6062 users. Of these, 4851 tests were applied in the Metropolitan Region and 1211 in the municipality of Coquimbo, between December 2011 and May 2012. The qualitative information was collected from semi-structured interviews and focus groups.

**Results:** The SBIRT program was perceived as more complete and standardized than treatment as usual in PHC for the problem of substance use. It is a program of relatively low complexity for health personnel, who were familiar with activities of this type. The times of application of the test and of the Brief Intervention are less compatible with the structure of work for certain PHC professionals, such as doctors, nurses, and midwives, unless it was divided into stages, with the detection phase performed by health technicians, and then intervention by professionals.

**Conclusions:** These findings shade light about strengths and necessary adaptations of the SBIRT program for widespread implementation.

## A40 Preliminary results of a study on the effectiveness of normative feedback as an active ingredient ofa brief intervention to reduce alcohol consumption among university students in Argentina

### Paula Victoria Gimenez, Karina Conde, Aldana Lichtenberger, Raquel Peltzer, Mariana Cremonte

#### Grupo de Sustancias Psicoactivas y Lesiones por Causa Externa, Facultad de Psicología, Universidad Nacional de Mar del Plata, Mar de Plata, Argentina

##### **Correspondence:** Paula Victoria Gimenez (gimenezpv@hotmail.com)

*Addiction Science & Clinical Practice* 2018, **13(Suppl 1)**: A40

**Background:** To evaluate the effectiveness of Normative Feedback (NF) in the Brief Intervention (BI) to reduce alcohol consumption and related problems in university students.

**Materials and methods:** Data were collected from 158 incoming students to the National University of Mar del Plata who provided informed consent. 60% were women and 40% were men, between 17 and 45 years old (M = 20.45 SD = 3.9). Weperformedd a random assignment to one of three conditions: evaluation only (control -CG-), evaluation and BI without RN (BI), and evaluation and BI with NF (BI-NF). To assess effectiveness, was evaluated the decrease in AUDIT scores. In order to estimate differences between the groups before and after the BIs, bivariate analyzes were performed (Kruskal–Wallis test and Mann–Whitney U test). Also measures of clinical significance (relative risk (RR), absolute risk reduction (RAR) and the number of patients needed to treat to reduce an event (NNT), comparing the effectiveness of interventions between groups.

**Results:** No differences were found between groups at the beginning of the experiment (H (2) = 3.52, p = 0.172), but they were found after interventions (H (2) = 25.6, p = 0.001). The initial means were CG M = 6.03, SD = 3.11, BI M = 5.24, SD = 2.41, and BI-NF M = 6.47, SD = 2.7; and the final CG M = 4.11, SD = 2.82, BI M = 1.92, SD = 1.53, and BI-NF M = 3.09, SD = 2.63. Differences were found between CG and BI (U = 688.5, p = 0.001), CG and BI-NF (U = 916.5, p = 0.018), and BI and BI-NF (U = 403.5, p = 0.013). The effectiveness was 85% CG, 97% BI and 91% BI-NF, the differences were significant for CG and BI (RR (IC95%) = 13% (21% -3%), RAR (IC95%) = 12% (2% -25%), NNT (IC95%) = 8 (4 to 39), p = 0.05), and CG and BI-NF [RR (IC95%) = 6% (19% -8%), RAR (95% CI) = 6% (-6% -22%), NNT (95% CI) = 18 (5 to -18), p = 0.04), although no BI and BI-NF differences were found (p = 0.248)].

**Conclusions:** Both analyzes showed the same tendency, the effectiveness of the BI, both without NF and with NF compared with the CG, but surprisingly the NF component seems not to contribute to the effectiveness of the BI.

## A41 Effectiveness of brief interventions to reduce alcohol consumption among older people in primary care: a review of systematic reviews

### Tassiane C. S. de Paula^1^, Danusa de Almeida Machado^1^, Maria Lucia O. Souza-Formigoni^1^, Emerita Opaleye^1^, Camila Chagas^1^, Cleusa P. Ferri^1,2^

#### ^1^Department of Psychobiology, Universidade Federal de São Paulo, São Paulo, Brazil; ^2^Health Technology Assessment Unit, Institute of Education and Health Sciences, Hospital Alemao Oswaldo Cruz, São Paulo, Brazil

##### **Correspondence:** Tassiane C. S. de Paula (tassiane.psi@gmail.com)

*Addiction Science & Clinical Practice* 2018, **13(Suppl 1)**: A41

**Background:** With the population aging, risks related to alcohol consumption among older people are a growing concern. However, studies on interventions to reduce alcohol consumption in older people are scarce.

**Materials and methods:** This is a review of systematic reviews on the effectiveness of brief interventions to reduce alcohol consumption among older people in primary care. The data sources were identified through the electronic databases: Medline, EMBASE and Cochrane, and through a search of the references of the texts identified in the search. Studies published in English prior to March 2018 were eligible for the review.

**Results:** Three recently published systematic reviews were identified: Kelly et al., (2017) included 13 studies, eight of which were conducted in primary care; Armstrong-Moore et al., (2018) included seven studies, six of which were in primary care; Kaner et al., (2018) included 69 studies, all in primary care with adult populations, but only four studies focused on older adults. 9 original studies were included in the systematic review, all of them conducted in high-income countries (HIC). The studies compared an intervention group (brief intervention based motivational interviews, personalized feedback, educational material, physician advice, drinking diaries and telephone counselling) and a control group (leaflets or usual care or no intervention). Five studies reported positive effects of the intervention on the reduction of alcohol consumption compared to controls, and the other four reported a reduction in harmful alcohol consumption that was not statistically significant. No evidence was found about the impact of these interventions on cognition or dementia, which are very relevant to aging.

**Conclusions:** Research in this area is still limited and concentrated in HIC. The studies adopted different approaches, which is positive in terms of identifying more effective interventions because they tested a wider range of options; however, their heterogeneous methods make comparisons difficult. Moreover, the interventions are not clearly described, making replication difficult. Future studies should focus on identifying which components of alcohol-related brief interventions proven to be effective for the adult population are also effective for the older population, and particularly those living in low- and middle-income countries.

## A42 Global prevalence of alcohol use during pregnancy and fetal alcohol spectrum disorder in the general population

### Svetlana Popova^1,2,3,4^, Shannon Lange^1,4^, Charlotte Probst^1,5^, Gerrit Gmel^1,6^, Jürgen Rehm^1,2,4,5^

#### ^1^Institute for Mental Health Policy Research, Centre for Addiction and Mental Health, Toronto, Canada; ^2^Dalla Lana School of Public Health, University of Toronto, Toronto, Canada; ^3^Factor-Inwentash Faculty of Social Work, University of Toronto, Toronto, Canada; ^4^Institute of Medical Science, University of Toronto, Faculty of Medicine, Medical Sciences, King’s College Circle, Toronto, Canada; ^5^Epidemiological Research Unit, Klinische Psychologie and Psychotherapie, Technische Universität Dresden, Dresden, Germany; ^6^School of Electrical Engineering and Telecommunications, Faculty of Engineering, The University of New South Wales, Sydney, Australia

##### **Correspondence:** Svetlana Popova (lana.popova@camh.ca)

*Addiction Science & Clinical Practice* 2018, **13(Suppl 1)**: A42

**Background:** Alcohol use during pregnancy is the direct cause of Fetal Alcohol Spectrum Disorder (FASD). To date, most countries do not have population-level prevalence data for alcohol use and binge drinking during pregnancy or Fetal Alcohol Syndrome (FAS)/FASD. Therefore, this was the first comprehensive epidemiological study to estimate the actual (based on existing empirical studies) and predicted (for countries with one or no empirical studies) prevalence of these indicators by country, WHO region, and globally.

**Materials and methods:** The methods included a comprehensive, systematic literature search; meta-analyses, assuming a random-effects model for countries with published studies; and regression modelling (data prediction) for countries with one or no studies. Estimated WHO regional and global averages of FAS/FASD prevalence were weighted by the number of live births in each country.

**Results:** This study has estimated that globally, about 10% of women consume alcohol during pregnancy and approximately eight out of 1000 children and youth have FASD in the general population. In some regions (most notably in the WHO European Region) a high proportion (about a quarter) of pregnant women in the general population consume alcohol during pregnancy, which is mirrored by also having the highest FASD prevalence (19.8 per 10,000; 95% CI 14.1–28.0) that is 2·6 times higher than the global average. In countries of the WHO Eastern Mediterranean Region and South-East Asia Region, where the rates of abstinence are very high, the prevalence of alcohol use during pregnancy and FAS/FASD was estimated to be the lowest.

**Conclusions:** Alcohol use during pregnancy is common in many countries. The prevalence of FASD in the general population exceeds one percent in 76 countries of the world, which is greater than the prevalence of some common birth defects. This highlights the need for more effective universal prevention strategies such as establishing a public health message about the detrimental consequences of prenatal alcohol exposure and a routine screening protocol. Brief interventions should be provided, where appropriate.

## A43 How many people with Harmful Alcohol Use are benefiting from face-to-face interventions in Chile?

### Paula Margozzini^1^, Nicolas Barticevic Lantadilla^2^

#### ^1^Department of Public Health, Pontificia Universidad Catolica de Chile, Santiago, Chile; ^2^Department of Family Medicine Pontificia Universidad Católica de Chile, Santiago, Chile

##### **Correspondence:** Paula Margozzini (pmargozzini@gmail.com)

*Addiction Science & Clinical Practice* 2018, **13(Suppl 1)**: A43

**Background:** Face-to-face interventions to reduce harmful alcohol use (HAU) have been encouraged by public policy in Chile; however, its coverage is unknown. Objective: Estimate and describe the national coverage of preventive and therapeutic interventions for HAU.

**Materials and methods:** The base year for the estimate was 2014. Care from the public treatment network (SENDA and Primary Care), treatment under the explicit guarantee scheme (GES) public and private, and the national program of brief interventions (BI) in primary care was included. A user in need of intervention was defined as having an AUDIT of 8 or more. The population in need of intervention was estimated using projections from the National Institute of Statistics and the prevalence of HAU according to the National Health Survey 2010.

**Results:** The prevalence of HAU is 12% (1,740,000 people) among those older than 15 years, and 131,373 subjects received face-to-face interventions: 58,925 were BI, and 72,448 some type of treatment. The national coverage for consumption at moderate risk of alcohol (AUDIT 8–15) was estimated at 5% and for problematic use or dependence (AUDIT 16 or more) 4%.

**Conclusions:** Despite considerable implementation efforts, the coverage for face-to-face preventive or therapeutic interventions for HAU in Chile remains low. 45% of these attentions correspond to BI, aimed at the people with the lower risk of damage within the HAU spectrum. Given the difficulty and costs of implementing a BI program, it is necessary to focus its growth towards a population with high multi-morbidity (e.g., patients of the cardiovascular program). The regulatory measures to discourage the consumption of alcohol in the entire population are essential as a public health measure.

## A44 Stigma toward drug users: a new Brazilian brief protocol for healthcare providers

### Joanna Gonçalves de Andrade Tostes^1^, Pollyanna Santos da Silveira^2,3^, Telmo Mota Ronzani^1,2^

#### ^1^Center for Research, Intervention and Evaluation on Alcohol and Drugs (CREPEIA), Federal University of Juiz de Fora, Sao Paulo, Brazil; ^2^Department of Psychology, Federal University of Juiz de Fora, Sao Paulo, Brazil; ^3^Department of Psychology, Catholic University of Petrópolis, Petrópolis, Rio de Janeiro, Brazil

##### **Correspondence:** Joanna Gonçalves de Andrade Tostes (joanna@tostes.org)

*Addiction Science & Clinical Practice* 2018, **13(Suppl 1)**: A44

**Background:** Drug use is considered one of the most stigmatizing health conditions. Evidence has shown that it is associated with several impairments as poor access to health care, low education levels, and unemployment. Stigma toward drug users is also found among healthcare providers. Besides the poor availability of services, many people who might benefit from treatment do not seek it or leave it prematurely to avoid stigmatization.

**Materials and methods:** We have developed an original brief intervention protocol. The proposed model, in a workshop format, is based on international approaches to changing the stigma and the Key Ingredients of Anti-Stigma Programs for Health Care Providers, developed by Mental Health Commission of Canada (MHCC). In addition, we have used the Acceptance and Commitment Training (ACT) approach. Among the several resources that have guided us, we highlight the ones available for members of the Association for Contextual Behavioral Science (ACBS), those that were sent by MHCC members, and the protocol sent by one founder of ACT. The workshop is composed by two sessions with a one week break. Each one lasts about six hours. Some of the strategies used allow participants to: understand how judgments are automatic and related to the stigmatizing process; observe parallels between reactions to drug users and their own psychological struggles; discuss what barriers they face in workplaces; consider the costs of internalizing stigma; analyze the attempts to control and avoid negative thoughts and emotions about users, about themselves, as well as their consequences and low effectiveness; identify their values and how far they are from them; and recognize ways to act more committed with these values, especially in the drug context.

**Results:** Now we are finishing the pilot process. We had to make some changes and adaptations, but we have maintained the largest majority of activities proposed. This protocol seems to be technically feasible and logistically viable. Now we will begin the implementation process in mental health services in our local area, and we will evaluate its indicators of effectiveness.

**Conclusions:** We need to move forward by implementing evidence-based strategies to reduce professional stigma indicators as this brief intervention.

